# Hitting the right target: taxonomic challenges for, and of, plant invasions

**DOI:** 10.1093/aobpla/plt042

**Published:** 2013-09-19

**Authors:** Petr Pyšek, Philip E. Hulme, Laura A. Meyerson, Gideon F. Smith, James S. Boatwright, Neil R. Crouch, Estrela Figueiredo, Llewellyn C. Foxcroft, Vojtěch Jarošík, David M. Richardson, Jan Suda, John R. U. Wilson

**Affiliations:** 1 Institute of Botany, Department of Invasion Ecology, Academy of Sciences of the Czech Republic, CZ-252 43 Průhonice, Czech Republic; 2 Department of Ecology, Faculty of Science, Charles University in Prague, Viničná 7, CZ-128 44 Prague, Czech Republic; 3 The Bio-Protection Research Centre, Lincoln University, PO Box 84, Canterbury, New Zealand; 4 Department of Natural Resources Science, University of Rhode Island, 1 Greenhouse Road, Kingston, RI 02881, USA; 5 South African National Biodiversity Institute, Biosystematics Research and Biodiversity Collections Division, Private Bag X101, Pretoria 0001, South Africa; 6 H. G. W. J. Schweickerdt Herbarium, Department of Plant Science, University of Pretoria, Pretoria 0002, South Africa; 7 Centre for Functional Ecology, Departamento de Ciências da Vida, Universidade de Coimbra, 3001-455 Coimbra, Portugal; 8 Department of Biodiversity and Conservation Biology, University of the Western Cape, Private Bag X17, Belville 7535, Cape Town, South Africa; 9 Ethnobotany Unit, South African National Biodiversity Institute, PO Box 52099, 4007 Berea Road, Durban, South Africa; 10 School of Chemistry, University of KwaZulu-Natal, Durban 4041, South Africa; 11 Department of Botany, Nelson Mandela Metropolitan University, PO Box 77000, Port Elizabeth 6031, South Africa; 12 Conservation Services, South African National Parks, Skukuza 1350, South Africa; 13 Centre for Invasion Biology, Department of Botany and Zoology, Stellenbosch University, Matieland 7602, South Africa; 14 Department of Botany, Faculty of Science, Charles University in Prague, Benátská 2, CZ-128 01 Prague, Czech Republic; 15 Institute of Botany, Laboratory of Flow Cytometry, Academy of Sciences of the Czech Republic, CZ-252 43 Průhonice, Czech Republic; 16 South African National Biodiversity Institute, Kirstenbosch Research Centre, Invasive Species Programme, Claremont 7735, South Africa

**Keywords:** Biological invasions, detecting new invasions, DNA barcoding, invasive plants, karyology, management, species identification, taxonomy.

## Abstract

This paper explores how a lack of taxonomic expertise, and by implication a dearth of taxonomic products such as identification tools, has hindered progress in understanding and managing biological invasions. It also explores how the taxonomic endeavour could benefit from studies of invasive species. We review the literature on the current situation in taxonomy with a focus on the challenges of identifying alien plant species and explore how this has affected the study of biological invasions. Biosecurity strategies, legislation dealing with invasive species, quarantine, weed surveillance and monitoring all depend on accurate and rapid identification of non-native taxa. However, such identification can be challenging because the taxonomic skill base in most countries is diffuse and lacks critical mass. Taxonomic resources are essential for the effective management of invasive plants and incorrect identifications can impede ecological studies. On the other hand, biological invasions have provided important tests of basic theories about species concepts. Better integration of classical alpha taxonomy and modern genetic taxonomic approaches will improve the accuracy of species identification and further refine taxonomic classification at the level of populations and genotypes in the field and laboratory. Modern taxonomy therefore needs to integrate both classical and new concepts and approaches. In particular, differing points of view between the proponents of morphological and molecular approaches should be negotiated because a narrow taxonomic perspective is harmful; the rigour of taxonomic decision-making clearly increases if insights from a variety of different complementary disciplines are combined and confronted. Taxonomy plays a critical role in the study of plant invasions and in turn benefits from the insights gained from these studies.

## Abstract (precis)

Taxonomic resources are essential for the effective management of invasive plants because biosecurity strategies, legislation dealing with invasive species, quarantine, weed surveillance and monitoring all depend on accurate and rapid identification of non-native taxa, and incorrect identifications can impede ecological studies. On the other hand, biological invasions have provided important tests of basic theories about species concepts. Modern taxonomy therefore needs to integrate both classical and new concepts and approaches to improve the accuracy of species identification and further refine taxonomic classification at the level of populations and genotypes in the field and laboratory.

## Introduction

The decline in taxonomic expertise substantially compromises rigorous studies in all fields of biodiversity or biogeography, including invasion biology (Smith *et al*. 2008*a*; Pyšek and Richardson 2010). Most regions of the world have been colonized by species from many other parts of the globe, and identifying many of these non-native species is a major challenge. Expertise in taxonomy (i.e. discovery, description and revision of taxa, and by implication determining the correct name of an organism) is crucial for implementing effective quarantine measures, monitoring invasions and their pathways, and ensuring that the time to first detection for new invaders is minimized. Additionally, resources are required for broad-based public participation in invasive species management and for providing information to horticulturalists, foresters and others who utilize, and often financially benefit from, alien species. Two conclusions of the Global Invasive Species Programme (GISP) were that ‘in most countries it will be found that more research will be needed on taxonomy and identification of species, and that there will often be a shortage of knowledge about natural distributions’ (Wittenberg and Cock 2001). We suggest that a decade after the completion of the first phase of GISP, this prediction has proved correct and the problem has reached critical proportions. More capacity in the taxonomy of plants and animals, both native and alien, is urgently needed (Smith *et al*. 2008*b*; Smith and Figueiredo 2009) because misidentification using both morphological and genetic data can have serious consequences. Furthermore, rapid identification of alien specimens can drastically reduce the time taken to respond to new or potential invasions.

A fundamental role of taxonomists in both taxonomic and non-taxonomic studies is to provide the correct scientific names by using the best available knowledge of the organisms submitted for identification. This service is provided to a range of clients—biologists, environmental managers, agronomists and environmental impact assessors—who require a robust framework of names in order to conduct their work accurately (Patterson *et al*. 2010). Despite the rapid advances in molecular techniques, classical (alpha) taxonomy is still useful and necessary in the 21st century. It is unlikely to lose this position since biology, especially biodiversity science, will always need taxonomists to do ‘real’ taxonomy (Godfray 2007), including the verification of the identity of organisms for which genetic data have been deposited into GenBank (i.e. garbage in, garbage out; see for example Shen *et al*. 2013). Additionally, many regions globally are unlikely to have easy access to such genetic data. In ecology, taxonomy is one of the fundamental units of currency (Gotelli 2004). The reliable identification of taxa (native or alien) paves the way for the study of organisms, potentially sheds light on many aspects of their biology, allows reference to the same taxa from other localities, and makes comparisons with congeners and other taxa possible. Incorrect identification of plant invaders as a result of inaccurate taxonomic services could lead to a misunderstanding of the dynamics of biological invasions. Moreover, alien species management is an international and multi-sectoral endeavour that requires accurate scientific names for global information sharing (Smith *et al*. 2008*a*).

This paper explores the ongoing and critical role of taxonomy in the study of plant invasions and specifically examines how the lack of taxonomic expertise can impede progress in understanding and managing invasions. The converse is equally true: we argue that taxonomy can also benefit from insights from biological invasions, a perspective that has not been sufficiently explored and emphasized in the literature. Finally, we suggest that classical taxonomy and modern genetic approaches must work in tandem, not only to improve the accuracy of species identification but also to potentially refine classifications at the levels of organism, population and genotype in the field and laboratory.

## Systemic Problems with Taxonomy

Is the current scope of taxonomy and nomenclature facing a crisis? Is the field of taxonomy suffering from a shortage of expertise and declining resources disproportionate relative to other disciplines in biology (Agnarsson and Kuntner 2007)? Or, are there now more taxonomists describing more species than ever before, as inferred from new databases showing that the number of taxonomists is increasing faster than the rate of species descriptions (Costello *et al*. 2013; but see Smith *et al*. 2008*b*)? Joppa *et al*. (2011) analysed the global rates of species descriptions, concluding that taxonomic research continues apace, despite contrary reports on the dissolution of taxonomic capacity. They determined that ‘the numbers of [flowering plant] taxonomists are increasing … as are the numbers of taxonomists who are the senior authors on species descriptions’. This led to their conclusion that ‘taxonomic description no longer belongs to those who do nothing else; species description is much more widely practiced’. However, there is a great difference between describing one new species and producing a taxonomic revision for a group of species, the former being a task often undertaken by amateurs whereas the latter demands greater perspective and most usually formal training and long-term employment.

Aside from these issues, it is clear that the field of taxonomy suffers from some systemic problems. Taxonomy is undervalued in current scientometric analyses (Krell 2000, 2002; Valdecasas *et al*. 2000; Agnarsson and Kuntner 2007; Seifert *et al*. 2008) and technological advances have provided new approaches to classifying biota, often at the expense of traditional approaches (Godfray 2002, 2005; Godfray and Knapp 2004; Godfray *et al*. 2008*a*, *b*). A primary reason why alpha taxonomy is undervalued in top-ranking journals is that such work is rarely hypothesis driven, is often of more local than global significance, applies standard rather than innovative approaches and has limited immediate impact on policy or management. Specialist taxonomic journals have relatively low impact factors, due to citation practices that include the convention of not citing original taxonomic descriptions or subsequent taxonomic revisions, the long citation half-life of taxonomic papers, regionality as a typical feature of taxonomic work, and because much important work in taxonomy is published in voluminous and immensely informative monographs rather than as succinct papers in journals. Furthermore, many authors cite papers that test the validity of taxonomic hypotheses with molecular data or papers that promote or use DNA barcodes, rather than papers based on classical taxonomy (Agnarsson and Kuntner 2007). The low number of specialists for particular groups of organisms further decreases the chances of taxonomic papers becoming highly cited (Krell 2002).

**Figure 1.**
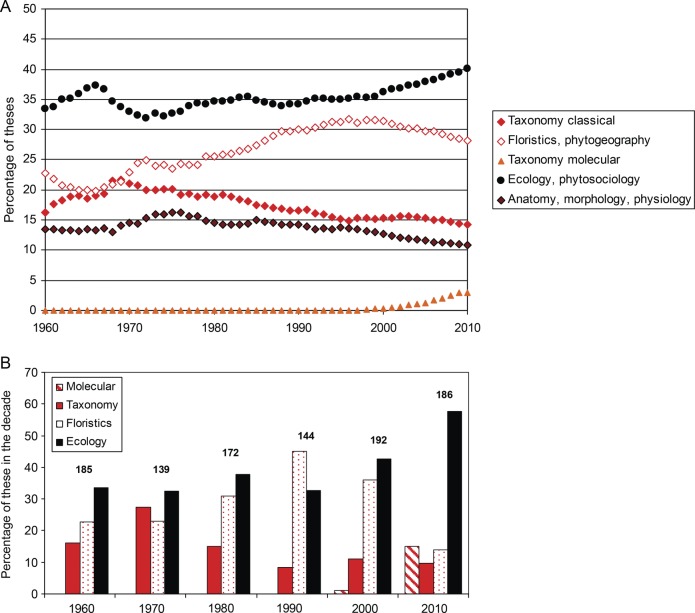
Trends in botanical interest at the Department of Botany, Charles University in Prague, Czech Republic over the last 50 years, expressed as the percentage of theses (master, doctoral, habilitation) in individual research fields (A), and by decades for areas relevant to plant invasion studies; those of interest for ‘taxonomy of invasions’, i.e. related to identification of alien species, are in red (B). Based on a total sample of 1018 theses completed in the examined period, with numbers for decades shown above bars.

In the sphere of education, experienced researchers formulate research topics for students and stimulate interest in taxonomy and other fields among future generations. Data gathered on 1018 theses completed from the 1960s to the present from the Department of Botany at Charles University in Prague, Czech Republic, a region where taxonomy has been traditionally very strong, allow us to examine how these trends translate into the interests of students and how research agendas shift over time (Fig. 1). The data reveal a steady, though slight, decline in the proportion of theses focusing on classical plant taxonomy between 1970 and 1990, after which this field maintained a low level (not exceeding 10 % of all theses completed in the department). Interestingly, molecular taxonomy seems to have overcompensated for the decline in classical plant taxonomy in the last decade, possibly indicating interest in a field that offers opportunities to do what is widely perceived as ‘cutting-edge’ science, with more attractive opportunities to publish in high-impact journals than is the case for classical taxonomy. On the other hand, the marked decrease in ‘floristics and phytogeography’ (from 36 to 14 % of all theses from the 2000s to the 2010s), which *inter alia* requires students to identify plants in the field, points to a declining interest in an area of expertise that is also highly relevant for invasion biology (Fig. 1). A similar declining trend in the number of students graduating with bachelor degrees in botany/plant biology has been reported from the USA between 1991 and 2008 (Kramer *et al*. 2010). Of particular concern is that the full impact of these trends is delayed as researchers work their way through the academic system. Fewer and fewer senior academics trained in classical taxonomy means fewer voices when it comes to setting institutional research agendas or departmental university priorities. This has the potential to create a negative feedback loop and general downward spiral in the advocacy required to maintain and promote taxonomy as highly relevant. Taxonomic expertise is rarely required when it comes to securing a job, especially in academia. Agnarsson and Kuntner (2007) estimated that as much as half the funding for taxonomic training may be lost due to lack of employer demand. Because the low profile of taxonomy results in serious underfunding in many parts of the world, employment opportunities are reduced for natural scientists, thereby further reducing the profile of taxonomy.

## Problem of Taxonomy for Biological Invasions

As in other fields of biology, taxonomic expertise in plant invasion biology is currently underfunded. For example, Europe suffers from a scarcity of experts particularly for insect and plant invasions (Hulme *et al*. 2009). This dearth of experts contrasts sharply with the recognition that taxonomists are increasingly needed to address the threats of biological invasions and that training opportunities and employment prospects for these skills are crucial at all career levels (Wheeler *et al*. 2004; Agnarsson and Kuntner 2007). Below we discuss problems related to the identification of newly arriving alien species, including the use of molecular tools, and examine how potential biases resulting from misidentification of alien species could impede the progress in ecological research on plant invasions.

### Accurate identification of species: detecting new invasions

The accurate identification of an organism under study is pivotal to all ecological research. The challenges faced by researchers and managers working on alien plant taxa are, however, often different from those faced by researchers who work on native species; the latter can accumulate considerable experience on the taxonomy of their group of specialization for their ‘patch’ (floristic province, state, country, region). Invasion ecologists usually do not have a specific biogeographic focus and require taxonomic information from much larger areas (essentially the whole world) for many taxa. Taxonomists specializing on a specific area rely on regional literature such as Floras (note the capital letter to denote published work) and identification keys that they can become well acquainted with during their careers. Such taxonomists are frequently able to flag taxa as alien (i.e. not represented in their regional floras). However, regional taxonomists may prove of little help if their expertise is geographical rather than linked to a particular taxonomic group. An increasing number of online taxonomic databases, such as the Annual Checklist of World Plants (www.sp2000.org), the International Plant Names Index (IPNI) (www.ipni.org), e-Floras (www.efloras.org) and the Germplasm Resources Information Network (www.ars-grin.gov), can be of some help to regional taxonomists but there is still a level of taxonomic expertise necessary to interpret such information, especially in specialized databases such as IPNI. The problem is especially pronounced in species-rich taxonomic groups that supply the highest number of invasive taxa such as plants (Pyšek *et al*. 2008). For example, 54 % of the 10 771 alien species recorded in Europe are plants (DAISIE 2009).

Current knowledge on biological invasions is geographically and taxonomically biased toward more developed regions, further complicating accurate species identification. Some regions, such as Asia, South America and Africa (excluding South Africa), are seriously understudied in terms of ecology (Pyšek *et al*. 2008). Since research intensity is generally related to economic prosperity (Leimu and Koricheva 2005), the same regions are also likely to be taxonomically understudied (Stuessy and Lack 2011), which is ironic since they are home to most of the world's biodiversity.

Historical information on alien species composition and distribution comes from floristic literature, herbaria and museum collections, seed suppliers and garden catalogues. Taxonomists are therefore well placed to track the introduction history of alien taxa via the preserved collections that they curate (Fuentes *et al.* 2008; Aikio *et al.* 2010). Scientific curation by taxonomists in herbaria requires constant and ongoing updating, especially for nomenclature and incorporating taxonomic changes. These collections are indispensable resources to facilitate locating alien species in the exact areas where they were initially collected and the habitats in which they were first described (Aikio *et al.* 2012). As in the case of requiring accurate species identification, expanding preserved collections (typically herbaria and natural history museums) of alien taxa is important as they provide both temporal-historical and accurate geographical information on such provenances for morphological and genetic data. These data also provide important historical information in cases where the alien population from which a specimen was gathered is subsequently cleared.

Identifying the origin of a species, i.e. determining whether it is alien to a given region, is closely associated with correct species identification. This can be complicated by species being native and alien in different parts of the same country (e.g. many species of *Acacia* in Australia: Bean 2007; Richardson *et al*. 2011; or *Spartina* in North America: Daehler and Strong 1994; Anttila *et al*. 1998) or continents (Lambdon *et al*. 2008), and by difficulties in distinguishing relatively recent natural dispersal events from human-mediated introductions (for examples from Antarctica: see Hughes and Convey 2012). Problems also arise from different taxonomic approaches in the native and invaded ranges. For example, the genus *Oenothera* has a specific reproduction system (permanent translocation heterozygosity) resulting in rapid formation of new species, which makes it difficult to align taxa invasive in Europe to their native North American counterparts, partly because some of them originated in the invaded range (Cleland 1972; Dietrich *et al*. 1997; Mihulka and Pyšek 2001).

### DNA barcoding and plant invasions

The last decade has seen substantial effort towards consolidating a fragmented taxonomic knowledge base through the use of web-based tools (Godfray 2002; Godfray *et al*. 2008*a*; Clark *et al*. 2009). The plea for web-based unitary taxonomy reflects the threat imposed on classical taxonomy by increasingly classifying biodiversity using available genetic sequence data. This trend therefore suggests that current taxonomy must embrace and absorb new trends rather than set itself in opposition to them (Godfray *et al*. 2008*b*). Such is the current prominence of molecular systematics that some journals (e.g. *Phytotaxa*) strongly discourage authors from attempting to publish papers that recognize classically determined families not accepted as valid by the Angiosperm Phylogeny Group (2009); the presentation of alternative family concepts otherwise requires a written justification (see e.g. http://www.mapress.com/phytotaxa/author.htm).

Taxon identification using standardized DNA gene regions or barcodes, i.e. DNA barcoding, is a rapidly developing research discipline with many strengths and possibilities (CBOL Plant Working Group 2009; Hollingsworth 2011). A DNA barcode can overcome several limitations of morphology-based taxonomy, including detection of morphologically cryptic species, recognition of species with high phenotypic plasticity, and individuals in early ontogenetic stages or incomplete and poorly developed specimens (Valentini *et al*. 2008). Molecular taxonomy can be particularly useful in groups that have received inadequate taxonomic attention (e.g. to reliably assess the diversity they contain) and it can enhance an understanding of species limits in groups with simple morphologies (serving as an independent arbiter between competing taxonomies). In general, DNA barcoding can modernize and revitalize conventional taxonomy and attract new specialists to this field, but ‘the promise of barcoding will be realized only if based on solid taxonomic foundations’ (Meyer and Paulay 2005; Godfray *et al*. 2008*b*). The names of organisms are linked to type specimens and the interpretation of these along with the correct nomenclature cannot be replaced by any molecular means.

DNA barcoding has recently been successfully applied in plant invasion biology. For example, this methodology has efficiently distinguished invasive aquatic species belonging to the genera *Cabomba*, *Ludwigia*, *Myriophyllum* and to the family Hydrocharitaceae from their non-invasive related counterparts (Ghahramanzadeh *et al*. 2013). Similarly, DNA barcodes proved successful in the identification of invasive *Solanum* species, with practical implications for plant biomonitoring (Zhang *et al*. 2013).

Despite these encouraging results, barcoding of terrestrial plants faces several challenges and limitations (Chase and Fay 2009), particularly for closely related species in which restricted variation in barcoding markers limits reliable identification; some plant genera have even been found to be refractory to barcoding (Piredda *et al*. 2011). Another limitation of widely used plastid barcodes for taxonomic decision-making stems from their uniparental inheritance (maternal in most angiosperms, paternal in the majority of gymnosperms) resulting in interspecific crosses remaining unrecognized and identified as their plastid donor parent. This can introduce serious bias in invasion studies because hybrids can show greater invasion potential than their parental species (Ellstrand and Schierenbeck 2000, Table 1; see also Smith and Figueiredo 2007 on a hybrid of *Agave* species in Portugal). Biparentally inherited nuclear barcodes might solve the problem and provide more information than organellar DNA. However, their value can be compromised by methodological issues and the presence of multiple divergent copies within a single individual. The risk of misidentification due to paralogy-related problems is particularly high in polyploids and polyploidization is common among invasive plants (te Beest *et al*. 2012). The complex evolutionary history of polyploid species (Soltis and Soltis 1999) can easily lead to incongruence between morphological and molecular identification that can only be reliably resolved by an experienced taxonomist with a deep understanding of processes shaping the variation of the group under investigation.

**Table 1.** Examples of taxonomically challenging genera where ecological studies profited from a detailed taxonomic study.

Genus	Taxa	Results	References
*Centaurea*	*C. stoebe*	Diploids prevail in the native range and are often monocarpic, the invasive cytotype is tetraploid and predominantly polycarpic.	Treier *et al*. (2009)
*Fallopia*	*F. japonica*, *F. sachalinensis*, *F.*× *bohemica*	Increased ploidy variation and rapid post-invasive evolution were observed in the invaded range. Particular genotypes of the hybrid taxon differ in invasiveness.	Mandák *et al*. (2003); Pyšek *et al.* (2003); Suda *et al*. (2010)
*Heracleum*	*H. mantegazzianum*, *H. persicum*, *H. sosnowskyi*	Taxonomic study disentangled overlapping distributions of congeners and the history of their introduction and indicated that the invasion occurred through multiple introductions.	Jahodová *et al*. (2007)
*Myriophyllum*	*M. sibiricum, M. spicatum*	Invasive populations resulted from hybridization of native and introduced species.	Moody and Les (2002)
*Phalaris*	*P. arundinacea*	Native and introduced populations have distinct genome sizes.	Lavergne *et al*. (2010)
*Phragmites*	*P. australis*	The introduced haplotype displaced the native one and invaded where the species previously had not occurred. Taxonomic study made it possible to disentangle the global pattern of the invasion.	Saltonstall (2002); Meyerson *et al*. (2010*a*, *b*, 2012); Lambertini *et al*. (2012); Meyerson and Cronin (2013)
*Rhododendron*	*R. catawbiense, R. ponticum*	Introgression from *R. catawbiense* increased the cold tolerance of invasive populations and allowed invasion to colder regions.	Milne and Abbott (2000)
*Spartina*	*S. alternifolia*, *S. anglica*, *S. maritima*, *S.* × *townsendii*	Hybridization of *S. alterniflora* with native *S. maritima* in the UK, and native *S. foliosa* on the West Coast of North America, resulted in formation of highly invasive types, including a new species *S. × townsendii* in the former region.	Anttila *et al*. (1998); Daehler and Strong (1994); Ayres *et al*. (2008); Ainouche *et al*. (2009)
*Tamarix*	*T. ramosissima*, *T. chinensis*, *T. parviflora*, *T. gallica*	Most invasive was a novel hybrid combination of two species-specific genotypes that were geographically isolated in their native range.	Gaskin and Schaal (2002)

### Biases in data for macroecological analyses due to the lack of taxonomic expertise

Much of the theory and current knowledge in plant invasion biology has arisen from analyses of secondary data, drawn from regional Floras, floristic literature and distribution atlases. Such analyses have made it possible to explore distribution patterns and invasion dynamics at various scales (e.g. Sax 2001; Pyšek and Hulme 2005; Cadotte *et al*. 2006; Wilson *et al*. 2007; Pyšek *et al*. 2009, 2010; Winter *et al*. 2009; Hulme 2011; Richardson *et al*. 2011). However, such databases and checklists can be seriously biased in terms of species present in a region and their distribution. Among the many types of errors that can plague occurrence databases, misidentification of species is arguably the most serious (Scott and Hallam 2002; Robertson *et al*. 2010; McGeoch *et al*. 2012), coupled with geographic and temporal variation in the nomenclature applied to a particular organism (Graham *et al*. 2004; Venette *et al*. 2010; Santos and Branco 2012). Groups for which the taxonomy is contentious or for those whose members are difficult to distinguish from one another are likely to be prone to the greatest bias (Ensing *et al*. 2013), including some invasive species (Rocchini *et al*. 2011). An additional problem of invasive species lists is inconsistent terminology (Hulme and Weser 2011; McGeoch *et al*. 2012) although detailed recommendations for standardization (e.g. Richardson *et al*. 2000; Pyšek *et al*. 2004; Blackburn *et al*. 2011) have been suggested and should reduce the problem in future.

A comparison of the alien flora of the Czech Republic (Pyšek *et al*. 2002) with data reported for that country in the *Flora Europaea* (Tutin *et al*. 1964–1980) provides quantitative insight into such biases. One hundred and eleven alien taxa naturalized in the Czech Republic (almost 50 % of the total number) were not reported in *Flora Europaea* for the Czech Republic at all (Pyšek 2003). This strong bias holds even if one accepts that this is a comparison of a specialized regional checklist with a source compiled for a continent several decades ago with no clear focus on alien species. The discrepancy in the number of naturalized species (present in the country for decades) is substantial.

Such biases seem to be due to insufficient research intensity coupled with a lack of taxonomic expertise. In the dynamic field of plant invasion ecology, focused taxonomic effort results in considerable improvement and almost invariably expansion of knowledge on organisms. For example, for the Czech Republic, a revision of a national checklist performed a decade after the original study yielded 151 taxa added to the list of alien plant species. These additions were due to detection of newly introduced species, new information due to investigation of sources omitted from the previous catalogue (including additional herbarium material), redetermination of previously reported taxa, reassessment of some taxa traditionally considered native and inclusion of intraspecific taxa previously not recognized in the flora. In addition, 134 names were changed for nomenclatural reasons or changes in taxonomic opinion (Danihelka *et al*. 2012; Pyšek *et al*. 2012*a*, *b*), 10 taxa were deleted because of being taxonomically unsound (hence now omitted from the Czech flora), and 16 were doubtful records previously only reported in the literature without herbarium evidence or taxa that were erroneously identified by the collector (Pyšek *et al*. 2012*a*, *b*). Similarly, 30 taxa were also omitted from the checklist of Belgian alien plants following a thorough taxonomic revision (Verloove and Lambinon 2008). Such challenges are not restricted to checklists of alien species and the magnitude of the bias usually only becomes obvious when modern monographs are published. This is illustrated by a comparison of data from modern monographs of Juncaceae and Potamogetonaceae with the 1997 IUCN Red List, which somewhat disturbingly was shown to be only correct for 20–25 % of species included (Kirschner and Kaplan 2002).

Nevertheless, much has recently been done in Europe to improve the situation with alien species checklists. The research conducted during the building of the DAISIE database illustrates the magnitude of taxonomic work behind any alien species database. With the DAISIE plant data, a merging of checklists of alien floras from the 48 regions considered, yielded 14 656 different names that had to be manually assigned to 5789 alien plant taxa reported for Europe in Lambdon *et al*. (2008); 48 taxa have been reported in local sources by 10 or more different names. Although the expertise needed to check and correct synonyms is not directly related to ability to identify species in the field, the above example shows that a huge taxonomic capacity is needed to build these databases and checklists, update and maintain them over time, and incorporate new data.

An additional concern is the historical under-representation of alien taxa in some herbaria. Of the 198 invasive species listed under South Africa's 2001 Conservation of Agricultural Resource's Act (CARA), 40 did not have herbarium records recorded in the country's National Herbarium (PRE) Computerized Information System (PRECIS) as of 2009, either because specimens were not collected or because data had not yet been uploaded (Wilson *et al*. 2013). Without physical specimens, it is impossible to verify the identity of invaders and update these data in the light of taxonomic changes or to collect DNA samples from archived material. In the case of South Africa, taxonomists have been appointed and provided with resources to facilitate this process specifically to detect new invaders before they become widespread (Wilson *et al.* 2013).

For macroecological analyses, such as those based on regional species richness (Pyšek *et al*. 2010; Essl *et al.* 2011), the implicit biases described above are less of a problem because even misidentified species still count toward the total number for a region regardless of their correct identity. Also, given the great differences in species richness across large regions such as Europe, slightly imprecise numbers are unlikely to affect the results of ecological studies significantly. For example, broad patterns showing the positive relationship between alien plant species richness and economic development in European countries are fairly robust to different sources of information (Hulme 2007; Pyšek *et al*. 2010). The same is likely to hold for studies that compare the species composition of whole alien floras across a large geographical range (Winter *et al*. 2009). Yet this robustness very much depends on using data that have used a standardized classification of species status. In a comparison of two major databases on alien species distribution in Europe, differences in nomenclature between the databases meant that interpretation of the correlates of alien species richness was dependent on the database used (Hulme and Weser 2011). These problems would be further accentuated in analyses that incorporate traits if these are incorrectly assigned to a species.

### When the lack of taxonomic expertise impedes progress in ecological research

The role of taxonomy therefore becomes explicit when determining the precise identity of species subject to detailed study. Examples of taxa that are taxonomically challenging include apomictic groups (e.g. *Crataegus*, *Pilosella*, *Rubus*, *Taraxacum*), karyologically variable complexes (e.g. *Centaurea, Fallopia*), genera with specific reproduction systems (e.g. *Oenothera*), or those for which horticulturalists have bred numerous cultivars and varieties (e.g. *Cotoneaster*) (Table 1).

The reliability of data for ecological studies can be limited in taxonomically or cytologically challenging species or complexes, particularly for historical data reported in the literature. For *Fallopia* taxa in the Czech Republic, sites reported in the literature and herbaria were revisited to explore whether the clones still persisted after decades since the first record. Redetermination of plants in the field revealed that 13 and 16 % of the records were misidentified for the two parental taxa, *Fallopia japonica* and *F. sachalinensis*. The misidentification rate was highest for the hybrid *F*. × *bohemica*, with 20 % of plants originally erroneously identified, either in the literature or as herbarium records, as one of the parental species (Pyšek *et al*. 2001). It was only after the complicated pattern of increased ploidy variation and rapid post-invasion evolution in the invaded range of Europe was disentangled that it was possible to carry out comparative ecological studies of the hybrid and/or its parents. For example, these studies documented increased competitive and regenerative ability and faster spread of the hybrid relative to the parents (Table 1).

*Phragmites* is an example of a genus where thorough taxonomic research in the last decade has provided insights into the global pattern of its multiple invasions. Three distinct lineages of *Phragmites australis* were identified in North America (Saltonstall 2002): (i) native *P. australis* subsp. *americanus* found throughout the USA and much of Canada; (ii) introduced *P. australis* subsp. *australis* from Eurasia found throughout North America; and (iii) the Gulf Coast lineage, *P. australis* var. *berlandieri*, found in the southern USA from Florida to California and extending into Central America. The origin of this lineage is unknown and it is therefore considered cryptogenic (Saltonstall 2002). However, the *Phragmites* story is further complicated by the hybridization of both the Gulf Coast type and the Eurasian type with other congeners, *P. karka* and *P. mauritianus* in the Gulf Coast region of the USA (Lambertini *et al*. 2012; Meyerson *et al*. 2012) that have produced a genetically and morphologically heterogeneous mosaic of *Phragmites* patches in this southern region. Most recently, a new introduced haplotype of *P. australis* (L1) was detected in Quebec, Canada, by sequencing the chloroplast DNA (Meyerson and Cronin 2013), suggesting that *Phragmites* diversity in North America may be increasing and that both molecular and morphological tools are needed to understand this rapidly evolving invasion.

Similarly, taxonomic advancement in the genus *Bolboschoenus* (Cyperaceae, formerly included in the genus *Scirpus*) achieved by classical methods during the last few decades has resulted in the reclassification of a taxon previously known as *B. maritimus*, with two subspecies in Europe (De Filipps 1980), into several closely related but distinct species well characterized by their morphology, karyology, ecology and distribution (Browning and Gordon-Gray 2000; Hroudová *et al*. 2007). Only then did it become obvious that populations introduced to eastern North America, where they became weedy in rice fields, belong to a distinct taxon, *B. glaucus* (Browning *et al*. 1995).

The *Fallopia* example mentioned above illustrates that identifying hybrids requires considerable taxonomic and karyological experience (Suda *et al*. 2010). There is an urgent need for progress in this area, as much evidence has emerged that hybridization regularly stimulates invasiveness, where the new taxon is more invasive than either parent (Abbott 1992; Vilà and D'Antonio 1998; Vilà *et al*. 2000; Abbott *et al.* 2003; see Ellstrand and Schierenbeck 2000 for a review and examples) or when it can invade new environments. *Spartina anglica*, a hybrid taxon that arose through allopolyploidization, is the classic example (Thompson 1991; Ainouche *et al*. 2009). With the emergence of karyological and molecular methods it has become obvious that invasiveness is often manifested below the species level, for example, at the cytological level (e.g. *Centaurea stoebe*—Treier *et al*. 2009) or in genotypes (*Fallopia* × *bohemica*—Pyšek *et al*. 2003; *Myriophyllum*—Moody and Les 2002; *P. australis*—Saltonstall 2002; Lambertini *et al*. 2012; Meyerson *et al*. 2012; *Rhododendron ponticum*—Milne and Abbott 2000; *Tamarix*—Gaskin and Schaal 2002). Besides the formation of new hybrid taxa, introgression more often results in hybrid swarms or in ‘genetic pollutions’, which are best examined at the gene level (Petit 2004).

Taxonomic uncertainty can also impede the results of ecological studies. For *Pilosella glomerata*, a European species invasive in North America, ecological niche models yielded varying predictions of its invasion potential depending on which genetic entities are used in training models (all records compared with only taxonomically verified records). Using all records resulted in substantially larger predicted potential ranges in the adventive range (Ensing *et al*. 2013). A similar result was reported for the Australian tree *Acacia saligna*, which is invasive in many parts of the world. Very different predicted ranges emerged from models trained using different genetic entities (tentatively subspecies) (Thompson *et al*. 2011). These are examples of rare empirical studies showing that vetting occurrence records for taxonomic reliability is crucial for niche modelling—and indeed for ecological research on invasions in general. Records of questionable taxonomic accuracy should be used with caution in ecological studies (Jimenez-Valverde *et al*. 2010).

### Risk of karyological bias introduced by species misidentification

**Figure 2.**
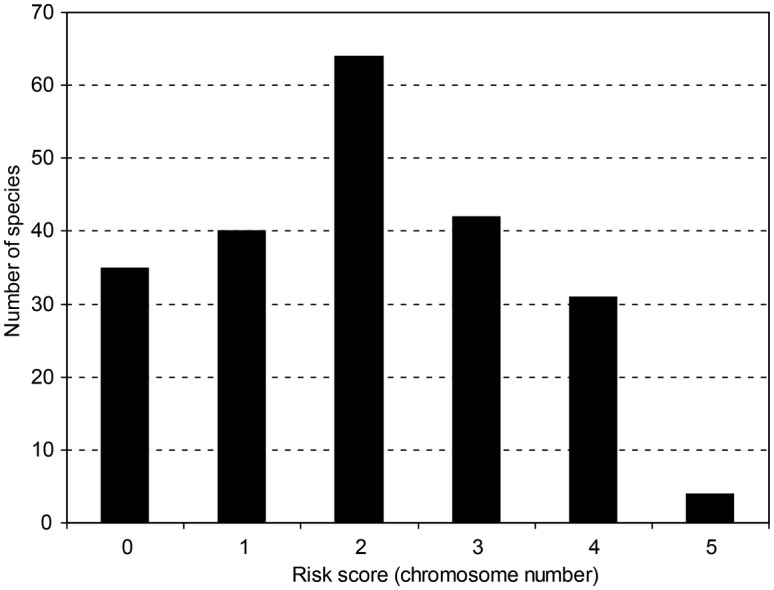
Frequency distribution of risk scores of introducing ploidy bias to ecological studies due to species misidentification, based on chromosome numbers of invasive plant species of the world and their non-invasive congeners (see Table 1 for delimitation of risk categories).

Karyology is a rapidly developing research area and genome characteristics are among the traits that have been recently used in studies explaining species invasiveness. Here as well, the lack of taxonomic expertise could affect the results of ecological studies. Evidence has accumulated in recent years that invasive behaviour can be associated with karyological characteristics, including variation in genome copy number (polyploidy) and genome size (te Beest *et al*. 2012). Polyploidization is one of the few mechanisms of instantaneous speciation and can rapidly alter organisms' traits by a single genetic event (Levin 2002). In species with multiple cytotypes, the polyploids are usually the ones that become invasive (te Beest *et al*. 2012). Similarly, at the interspecific level, polyploids are overrepresented among invasive aliens relative to native or rare species in local floras (Pandit *et al*. 2011). The high incidence of ploidy heterogeneity within plant genera entails the risk of ploidy mismatch: if the samples are not correctly assigned to species, incorrect ploidy levels can be introduced into a data set due to species misidentification, and the results of a study addressing the effects of karyological characteristics on invasiveness can be affected. We analysed data on ploidy variation from the *Index to plant chromosome numbers* (Goldblatt and Johnson 1979 onwards) in invasive land plant species of the world (as listed by Weber 2003) and their non-invasive congeners (not included in Weber's checklist). This analysis reveals that the risk of ploidy bias due to species misidentification is likely to be small in 75 of the 216 genera (34.7 %), and intermediate in 106 genera (49.1 %; Appendix1; Fig. 2). More importantly, the danger of ploidy mismatch between globally invasive and non-invasive species is comparatively high in 35 genera (16.2 %), the most salient examples being the genera *Amorpha*, *Chromolaena*, *Litsea* and *Syzygium*, in which the ploidy levels of species from both groups are mutually exclusive.

Inherently associated with ploidy is the amount of nuclear DNA (genome size). Genome size can constrain several characteristics that can underpin invasive success, including minimum generation time, seed mass, growth rate and specific leaf area (Leitch and Bennett 2007). A negative correlation between invasiveness and genome size was reported, for instance, in pines (Grotkopp *et al*. 2004) while Kubešová *et al*. (2010) showed that alien species naturalized in the Czech Republic have, on average, smaller genomes than their non-invading congeners. Because genome size often varies considerably even among closely related species, accurate species determination is an essential prerequisite for any studies addressing the role of this cytogenetic parameter. Using the same data set as above, we assessed the magnitude of risk that holoploid genome size values (taken from the Plant DNA C-values database; Bennett and Leitch 2012) would be biased if globally invasive species (Weber 2003) were misidentified with their non-invasive congeners. We found low and intermediate risk for 43 and 51 % of genera, respectively, whereas considerable differences between both species groups were revealed in seven out of the 109 genera with available data (6 %), including *Imperata* and *Vinca* (Appendix1).

## Problem of Biological Invasions for Taxonomy

Thus far we have focused on the critical role of taxonomy in studying and managing plant invasions. We now turn our attention to what the dynamic field of invasion biology has to offer to taxonomy. An increasing number of studies have demonstrated that processes such as evolution and speciation can occur rapidly in species that are introduced to novel ranges and become invasive. These almost ‘real time’ developments offer exciting opportunities to refine taxonomic approaches to better address the novel species assemblages that are associated with rapid global change.

The concept of ‘species’ as the basic category of biological classification is fundamental to taxonomy and the importance of species in biology derives primarily from their importance in the taxonomic framework used in all branches of biology (de Queiroz 2005). Although the species concept has generated considerable controversy (Hey 2006) by distinguishing between species conceptualization (the evolutionary history of a lineage) and species delimitation (different properties acquired by lineages during the course of divergence), a unified concept has helped clarify the debate (de Queiroz 2007). A range of criteria have been proposed to support species delimitation (Table 2) and it is widely believed that the presence of any one of the properties (if appropriately interpreted) is evidence for the existence of a species, though more properties and thus more lines of evidence are associated with a higher degree of corroboration. While the different species concepts have faced various challenges, alien plants are increasingly forcing taxonomists to rethink the concepts they apply to ‘species’.

**Table 2.** Common species concepts, their definition and examples of challenges posed by alien plant species.

Species concept	Definition	Challenges
Phenetic species concept	A species is a set of organisms that are phenotypically similar and that look different from other sets of organisms.	Misidentification of the introduced *U. pertusa* as native taxa *U. rigida*, *U. pseudocurvata* or *U. scandinavica* delaying any action against this potentially invasive species (Baamonde *et al.* 2007).
Biological species concept	A species is a group of individuals that can breed together but cannot breed with other groups.	Numerous examples of distantly related native–alien plant crosses resulting in an invasive hybrid (Schierenbeck and Ellstrand 2009).
Recognition species concept	Species are the most inclusive population of individual biparental organisms, which share a common fertilization system.	Allopolyploidy and clonality can stabilize lineages resulting from native–alien crosses that would normally suffer hybrid sterility (Schierenbeck and Ellstrand 2009).
Phylogenetic species concept	A species is a single lineage of ancestral descendant populations or organisms that maintains its identity from other such lineages and that has its own evolutionary tendencies and historical fate.	Invasive variable leaf watermilfoil (*M. heterophyllum*) in the northeastern USA consists of at least three distinct lineages: an interspecific hybrid and two historically allopatric lineages (Tavalire *et al.* 2012).
Genotypic cluster species concept	A species is a morphologically or genetically distinguishable group of individuals that has few or no intermediates when in contact with other such clusters.	Only after the marked expansion of *P. australis* in North America have the native and introduced lineages of common reed been designated as distinct subspecies (Saltonstall *et al.* 2004).
Ecological species concept	A species is a lineage (or a closely related set of lineages) that occupies an adaptive zone minimally different from that of any other lineage in its range and that evolves separately from all lineages outside its range.	Species occupy new ranges/habitats in their invasive ranges.

At its simplest, taxonomists attempt to identify and distinguish species based on reproducible and consistent morphological criteria. The phenetic species concept defines a species as a set of organisms that look similar to each other and distinct from other sets, usually assessed through a multivariate phenetic distance statistic derived from measures of many characters across many organisms. However, the invasion of alien species that are morphologically indistinguishable, or hardly distinguishable, from native species or earlier established species is regarded as a cryptic invasion (Chu *et al.* 2012). For example, species-level identification in the seaweed genus *Ulva* is typically difficult, notably in view of the intraspecific variability often seen in the rather few morphological and anatomical characters used for species discrimination. *Ulva pertusa* is widely distributed in the Indo-Pacific Ocean but has been introduced with shellfish aquaculture to the Atlantic coast of Europe. On the NW Iberian Peninsula coast *U. pertusa* has previously been misidentified as native taxa *Ulva rigida*, *U. pseudocurvata* or *U. scandinavica*, thus delaying any action against this potentially invasive species (Baamonde *et al.* 2007).

Morphology on its own may therefore not be a reliable guide to a species; thus the biological species concept describes a species as a group of individuals that can breed together but cannot breed with other groups, independent of morphological similarity. While hybridization in the wild between closely related species is not unusual, the global interchange of species between different regions of the world challenges the biological species concept. Many hybridization events are recorded, either between native and alien taxa or between alien taxa brought together for the first time in a novel region (Ellstrand and Schierenbeck 2000; Schierenbeck and Ellstrand 2009). Interspecific hybridization can result in genetic swamping of native taxa or an increase in colonization probabilities and rates (Hovick *et al.* 2012). For evolutionarily well-differentiated taxa, reproductive isolating barriers are often strong and the resulting hybrids are sterile. However, the study of biological invasions has shown how even where reproductive isolating barriers may be strong, new taxa can occur following hybridization. Allopolyploidy and clonality can stabilize lineages that would suffer sterility as F1 hybrids while ﬁxing hybridity and novelty (Schierenbeck and Ellstrand 2009).

The ability of perennial plants to persist and spread clonally in the absence of seed production provides a powerful mechanism to overcome even strong fertilization barriers and runs counter to the recognition species concept. Indeed, where genetically distinct asexual clones are found for a particular taxon, then under either the biological species concept or the recognition species concept, such taxa could be described as separate species. *Bryonia alba* (Cucurbitaceae) is a herbaceous Eurasian vine that reproduces predominantly clonally (asexually) through apomixis, but possesses moderate to high levels of clonal diversity in its introduced range in the western USA—probably the result of multiple introductions and founder events (Novak and Mack 2000). In contrast, in New Zealand, the apomictic *Hieracium lepidulum* demonstrates high intra- and inter-population genotypic diversity as a result of both recombination and mutation (Chapman *et al.* 2004).

The challenge of asexual species has led to the consideration of a phylogenetic species concept where a species is a single lineage of ancestral descendant populations or organisms that maintains its identity from other such lineages and that has its own evolutionary tendencies and historical fate. However, alien plants challenge this assumption yet again in that hybrids may be found between quite distinct historical lineages. The invasive *Myriophyllum heterophyllum* in the northeastern USA comprises at least three distinct lineages: an interspecific hybrid (*M. heterophyllum* × *M. laxum*) and two historically allopatric lineages of pure *M. heterophyllum* (Tavalire *et al.* 2012).

Rather than focus on the evolutionary lineage, it has been proposed that perhaps a species should simply be distinguished by being a morphologically or genetically distinguishable group of individuals that has few or no intermediates when in contact with other such clusters. Two examples from the invasive species domain challenge this genotypic cluster species concept. *Rubus alceifolius* has been introduced to Indian Ocean islands where populations on each island are characterized by a single different genotype—the result of successive nested founder events leading to a cumulative reduction in genetic diversity (Amsellem *et al.* 2000). It remains controversial as to whether such genetically distinguishable clones should be treated as separate species.

In contrast, alien plants have highlighted how an existing taxonomic group may indeed comprise multiple genetically distinct subspecies. The invasive European genotype of *P. australis* rapidly outcompeted the native genotype in North America with the result that the distribution and abundance of European *P. australis* in North America have increased dramatically over the past 150 years (Saltonstall 2002). Marked differences in genetic structuring and population diversity have been found between the native and introduced lineages (Saltonstall 2002; Meyerson *et al*. 2009; Kettenring and Mock 2012) and some evidence for hybridization has begun to emerge (e.g. Meyerson *et al*. 2010*b*, 2012; Paul *et al*. 2010; Lambertini *et al*. 2012). Thus, what was believed to be a single species behaves, in terms of morphology and genetics, as two different species. It was only after the recognition of the two lineages in 2002 that they were designated as distinct subspecies, i.e. the North American native is subsp. *americanus* while the European lineage is subsp. *australis*. In the Gulf Coast of the USA an even more complex story of inter- and intraspecific hybridization among *Phragmites* species and lineages is occurring (Lambertini *et al.* 2012; Meyerson *et al*. 2012), further demonstrating that this genus is taxonomically more complicated than previously recognized. The *Phragmites* story illustrates how both classic and molecular taxonomy work in tandem.

A final example of how alien plant species can challenge species concepts is demonstrated by the ecological species concept where a species is a lineage that occupies an adaptive zone minimally different from that of any other lineage in its range and that evolves separately from all lineages outside its range. However, increasing evidence reveals situations where alien species occupy distinct niches in their introduced range relative to their native range (Guo *et al.* 2013; Hulme and Barrett 2013). Under these circumstances, such events may call into question the relevance of the ecological species concept. Where these niche shifts also include changes in genotype frequencies and morphological traits, these alien invaders may reflect the initial stage of speciation, especially if reproductive isolation follows as is the case for asexual species.

While the above is not an exhaustive assessment of species concepts (see also Table 2), it does illustrate that while the study of plant invasions requires a detailed understanding of taxonomy, invasive species pose considerable challenges, and many opportunities, to taxonomists.

## Implications for Management

Taxonomy is required for invasion biology beyond simply the challenges associated with cataloguing life, i.e. adding the name of an organism to a biological inventory. Failure to identify the invasive entity correctly or identifying it too late can result in a delayed response to a nascent invasion, or the application of inadequate or inappropriate management measures. In Belgium, the initial erroneous identification or confusion of four alien species (*Digitaria violascens*, *Eleocharis pellucida*, *Juncus dichotomus* and *Vicia dalmatica*) with non-invasive species made it possible for noxious environmental weeds to become invasive and a problem for conservation management (Verloove 2010). Because hybrid populations may respond differently to local ecological conditions than their parents (Thompson 1991; Milne and Abbott 2000), information on hybrid presence and distribution is also of particular importance to management and conservation programmes (Moody and Les 2002). Consequently, inadequate taxonomic resolution limits options for early detection and rapid response and for various subsequent management options when the wrong species is targeted by specific control measures.

Accurate identification is also critical for the classical biological control of invasive plants. For example, biological control of Cactaceae in South Africa was delayed because the wrong species of herbivorous cochineal insect was collected; after the taxonomic problems associated with the identification of *Cylindropuntia fulgida* var. *fulgida* were resolved and the appropriate insect was released, the population of the invasive plant declined (Paterson *et al*. 2011). Phylogeographic tools have helped such situations. For example, biocontrol researchers used a molecular approach to identify the specific origin of *Lygodium microphyllum*, a fern invading Florida. They then identified which haplotype of a phytophagous mite, *Floracarus perrepae*, was naturally associated with the invasive fern in Florida, and found that this mite was significantly more damaging than other haplotypes (Goolsby *et al*. 2006). Correct taxonomy also provides insights into the groups and geographic locations that should be searched for potential control agents in the weed's native range, and which plants in the introduced range should be included in host specificity assessment to determine the risks of non-target impacts (Wapshere 1974; Briese and Walker 2008). Finally, classical biological control is an example where applied needs in the invaded range stimulate and fast-track taxonomical work in the native range, e.g. the first formal description of many agents only occurs because the agents have promise for biological control.

In general, legislation explicitly relies on the biological species concept. However, we need to understand all aspects of invasions, and the biological species concept is inadequate for resolution of some key facets of invasion ecology such as the crucial details of subspecific differences, cultivars and hybrids. For example, horticulturalists and regulators need tools to separate non-invasive from invasive cultivars (Wilson and Hoch 2009). At a minimum, legal frameworks should support identification and monitoring of alien species below the species level as part of a broader requirement for identifying and monitoring components of biological diversity (Shine *et al.* 2000).

## Conclusions: The Way Forward

Failure to correctly identify an organism can lead to spurious conclusions and ultimately to inappropriate and ineffective legislation, management and policy tools, from the local to the global scales. The study of biological invasions needs to utilize and integrate ecological, molecular and morphological information on alien species to better understand particular invasions, and to inform appropriate management interventions. Specifically, it requires (i) field floristic knowledge for recording plants in the field and their distributions; (ii) traditional (alpha) taxonomy based on the ability to assess morphological samples; and (iii) molecular systematics using genetic tools to identify taxa and localize the specific origin of invaders. The latter two approaches are often combined, but all three areas are essential for improving the quality of invasive plant databases.

Unfortunately, taxonomy is undervalued in current scientometric analyses, which is reflected in poor funding opportunities in many parts of the world. A greater focus on education is required. A drawcard for taxonomy to attract students these days is the link with molecular work. Closer links between molecular work and what is often called classical taxonomy will also probably help to remedy the citation and impact factor issue as new classifications/taxonomy linked to studies of evolutionary relationships are likely to make taxonomic papers more competitive in the current scenario of citation practices. On the other hand, molecular studies will profit greatly from the deep insights and knowledge that can only be gained by a long-term natural history study of living plants in the field, such as are regularly undertaken on many invasive species.

Although DNA barcoding offers exciting new research possibilities (Cross *et al*. 2011), it is no panacea. Even in the genomic era automated identification of all plant species based on DNA markers is, and probably will remain, an unrealistic goal, making conventional taxonomy an irreplaceable discipline. Effectively addressing the current and future challenges of invasion biology requires collaborative taxonomic expertise from both classical and molecular approaches. A more ‘integrative taxonomy’ (Pires and Marinoni 2010) would combine the strengths of both traditional and molecular taxonomy and profit from their synergistic use, and potentially produce new tools for invasion biology and taxonomy alike. For example, molecular tools may assist us in detecting and identifying finer scale morphological differences in both genotypes and hybrids, thereby facilitating more rapid and accurate differentiation in the field and further enriching taxonomy overall.

The time has come for a resurgence and reinvestment in taxonomy for the 21st century and beyond. Global change (in the form of biological invasions and climate change) is creating novel environments in which plant communities are likely to respond in both fascinating and unexpected ways. Trained taxonomists are indispensable for understanding the changes in the Earth's biota, and for providing insights for management and conservation. Reliable taxonomic keys require data from both classical taxonomists and molecular biologists. Species recognition will continue to be a fundamental basis for all basic and applied biological research. In invasion biology, correct species identification and knowledge of interspecific diversity and traits determine the success and, consequently, cost of biological control programmes. For practitioners, reliable determination keys based on morphological characters will continue to be a major information source for species identification because the majority of non-specialists will never have access to molecular methods nor the skills to use them for distinguishing species.

## Sources of Funding

P.P., V.J. and J.S. were supported by grant no. P505/11/1028 (Czech Science Foundation), long-term research development project no. RVO 67985939 (Academy of Sciences of the Czech Republic) and institutional resources of the Ministry of Education, Youth and Sports of the Czech Republic. P.P. acknowledges support from the Praemium Academiae award from the Academy of Sciences of the Czech Republic. L.A.M. was supported by the US National Science Foundation DEB Award 1049914 and the University of Rhode Island College of Environment and Life Sciences Agricultural Experiment Station Project RH 00565. L.C.F. was supported by SANParks, and L.C.F., D.M.R. and J.R.U.W. by the DST-NRF Centre of Excellence for Invasion Biology, Stellenbosch University, and the National Research Foundation (grant 85417 to D.M.R.).

## Contributions by the Authors

P.P., G.F.S., D.M.R., J.S.B., N.R.C., L.C.F. and V.J. conceived the idea at a workshop at the Kirstenbosch National Botanical Garden, Cape Town, and a follow-up meeting at the Centre for Invasion Biology, Stellenbosch, in 2010. P.P., V.J. and J.S. collected the data, P.P., P.E.H., L.A.M., J.R.U.W. and J.S. wrote the paper and all authors commented on the manuscript.

## Conflict of Interest Statement

None declared.

## Appendix

**Appendix1.** Comparison of chromosome numbers and genome sizes among globally invasive plant species and their non-invasive congeners.

Genus	Chromosomes (invasive)	Chromosomes (non-invasive)	RISK (0–5)	Genome size (invasive)	Genome size (non-invasive)	RISK (0–2)
*Abrus*	22	24	1			
*Acacia*	26, 52, 104	26, 52, 78, 104, 208	2	0.55–1.78	0.53–2.10	0
*Acer*	26, (42), 52	26, 34, 52	1	0.54–1.35	0.38–1.35	0
*Agapanthus*	30	30	0	12.68	11.23–23.78	0
*Agave*	60, (90), 120, 150	60, 90, 120, 150, 180	1	3.05–11.31	2.98–12.53	0
*Ageratina*	51, 102	34, 51, 68,102	2			
*Agrostis*	28, 42	14, 28, 42, 56, 84	4	3.50–4.65	1.68–10.89	2
*Aira*	14, 28	14, 28	0	6.03	2.93–5.35	1
*Albizia*	26	26, (78)	1			
*Allium*	18, 27	14, 16, 18, 20, 21, 22, 24, 28, 30, 32, 35, 36, 40, 42, 46, 48, 64	2	18.15	7.60–74.50	2
*Alstroemeria*	16	16, (13, 25)	1	26.75–40.45	18.25–39.5	0
*Alternanthera*	100	28, 32, 34, 40, 96	4			
*Amorpha*	40	20	5			
*Andropogon*	20, 40	20, 30, 40, 60, 70, 80, 90	3			
*Annona*				1.35	0.68–1.43	0
*Anredera*	24, 36	24	2			
*Anthoxanthum*	10, 20	10, 20, 40, 56, 70, 86, 88	4			
*Aponogeton*				3.95	1.73	1
*Argemone*	28, 56	28, 56	0			
*Arrhenatherum*	(14), 28	14, 28	1	7.98	4.81	0
*Asparagus*	20, 40	16, 18, 20, 30, 40, 60	2	1.35–2.18	1.28–2.65	0
*Asphodelus*	28, 56	14, 28, 52, 56, 70, 84	3			
*Atriplex*				0.85	0.43–2.99	1
*Avena*	(28), 42	14, 28, 42	2	14.15	4.00–13.7	1
*Azolla*	(40), 44, 66	44, 48, 52, 66, 88	2			
*Berberis*	28	(24), 28, 42, 56	2	0.70–1.55	0.50–3.10	1
*Bidens*	48, 72	24, 32, 34, 36, 48, 60, 66/68, 72, 80, 84, 96	3	1.73	1.60–3.22	0
*Brassica*	20	16, 18, 20, 22, 24, 27, 28, 32, 34, 36, 38, 40	3	0.55	0.50–3.93	1
*Briza*	14	10, 14, 28	2	6.48	2.90–11.68	1
*Bromus*	14, 28, (42), 56	14, 28, 42, 56, 70	1	3.25–12.27	1.88–16.33	0
*Buddleja*	76	38, 76	2	1.37	0.86–0.94	1
*Caesalpinia*	22, 24	24, 48	2			
*Calotropis*	22	22	0			
*Cardiospermum*	20, (22)	14, 20, 22	1			
*Carduus*	16, 22, 32, 54, 64	16, 18, 20, 22, 24, 26, 28, 30, 32, 34, 36, 54, 62	1			
*Casuarina*	18	18	0			
*Cecropia*	28	26, 28	1			
*Celastrus*	46	46	0			
*Celtis*	20	20, 30, 40	2			
*Cenchrus*	32, 34, 36, 54, (72)	34, 36, 42, 45, 48, 102	2	1.33	2.55–5.56	1
*Centaurea*	16, 18, 20, 36	14, 16, 18, 20, 22, 24, 26, 27, 28, 30, 32, 34, 36, 40, 42, 44, 48, 50, 52, 54, 60, 66, 84, 90, 110	3	0.87–1.57	0.83–2.15	0
*Cestrum*	16	16	0			
*Cinnamomum*	24	24	0			
*Cirsium*	34, 68	20, 21, 22, 23, 24, 26, 28, 30, 32, 34, c. 60, 68, c. 102	3	1.42–2.77	1.07–1.8	0
*Clematis*	16	16, 32, 48	3	9.05	9.65–15.80	1
*Clidemia*	34	20, 34, 46, 48	3			
*Colocasia*	28, 42	28, 42, 44, 46, 52, 58, 116	4	4.08	3.33	0
*Conium*	22	22	0			
*Coprosma*	44	22, 44, 88, 132, 220, 264	4	1.38	1.28	0
*Cortaderia*	72	90	2			
*Cotoneaster*	68	34, 51, 68	2	1.37	0.71–1.39	0
*Cotula*	20, 40	18, 20, 36, 52, c. 80, 104, 156, 208, 260	4			
*Crassula*	14	14, 16, 32, 42, 48, c. 56, 64, c. 84, c. 90, c. 96	4			
*Crataegus*	34, 51	24, 34, 48, 51, 64, 68, 72	3	0.76	0.63–1.77	1
*Cynara*	34	34	0			
*Cynodon*	18, 27, 36, 54	18, 36	1	0.80–1.47	0.52	1
*Cyperus*	42	16, 18, 22, 24, 26, 30, 32, 34, 36, 38, 40, 44, 46, 48, 50, 52, 54, 56, 58, 64, 68, 70, 72, 74, 78, 80, 82, 88, 96, 108, 112, 134, 136, 152, 186, 208, 220, 224	4			
*Cytisus*	46, 48	24, 46, 48, 50, 52, c. 84, 96	4	0.85	0.55–2.07	1
*Dactylis*	14, 28	14, 28	0	3.30–4.40	2.16–2.26	1
*Datura*	24	24, (36), (48)	1	2.05	1.73–2.28	0
*Dioscorea*	40, 50, 60, 70, 80	20, 30,(36), 40, (56), 60, (64), 70, 80, 100, 120, 140	3	0.58–1.06	0.35–6.75	1
*Dipsacus*	18	16, 18, 36	2	3.28–3.21	3.29–5.29	0
*Egeria*	46	52	1			
*Ehrharta*	24, 48	24, 48, 72, 96, 120	3			
*Echinochloa*	108	18, 36, 48, 54, (56), (108)	4			
*Echium*	16	12, 14, 16, 24, 32	2			
*Eichhornia*	32, 64	16, 30	2			
*Elaeagnus*	28	28	0			
*Elodea*	24, 32, 40, 48, 56, 64, 72, 96	24, 48	1			
*Elytrigia*	28, 35, 42, 49	24, 28, 35, 42, 56, 63	1	11.64–17.46	13.25–20.28	0
*Eragrostis*	20, 30, 40, 50, 60, 70, 80	20, 30, 40, 50, 60	1			
*Erica*	24	24	0			
*Erigeron*	(27), 36	18, 27, 36, 45, 54	3			
*Erodium*	20, 40, 60	16, 18, 20, (27), 36, 40, (56), 60	1			
*Eucalyptus*	22	22, (44)	1	0.55	0.39–0.74	0
*Eugenia*	22, 33	22, 44, 66	2	0.25	0.24–0.32	0
*Euphorbia*	16, 20, 60, (64)	10, 12, 14, 16, 18, 20, 22, 24, 26, 28, 30, 32, 36, 40, 42, 44, 46, 56, 60, 72, 80, 90, 120, 160	2			
*Fallopia*	44, 66, 88, 110	20, 22, 40, 44, 132	3	2.43–4.82	0.35–4.63	1
*Festuca*	28, 42, 56, 70	14, 21, 28, 30, 35, 42, 49, (52), 56, 70	2	4.28–8.49	1.94–12.84	1
*Ficus*	26	26	0	0.37	0.68–1.5	1
*Foeniculum*	22	22	0			
*Frangula*				0.33	0.34	0
*Freesia*	22	22	0			
*Fuchsia*	44, (120)	22, 44, 55, 88	4	1.47	0.73–1.72	1
*Galium*	64, 66	20, 22, 24, 40, 44, 48, 66, 88, 96, 132	4	1.03	0.51–1.89	1
*Genista*	44, 46, 48	(12), 18, (22), 24, (26), (27), 30, 32, 36, 40, 44, 46, 48, 50, 52, (54), (58), (60), 72, 88, 96, 120, 132	4			
*Gladiolus*	30	22, 24, 28, 30, 45, 50, 52, 54, 56, 58, 60, 80, 90 120, 170, 180	3			
*Gleditsia*	28	28	0			
*Glyceria*	20, 40, 56, 60	20, 28, 30, 40, 60	1			
*Gunnera*	34	34	0			
*Hedera*	48	48, 96, 144, 192	3	1.48	1.40–5.45	1
*Hedychium*	34	34, 68	2			
*Helianthus*	102	34, 68, 102	4	12.55	2.43–12.95	1
*Heracleum*	22	22, 40, 44	2	1.78	2.19	0
*Hesperis*	14, 24, 28	12, 14, 16, 28	1			
*Hiptage*	58	58	0			
*Holcus*	14	8, 14, 21, 28, 35, 42	4	1.7	2.78–4.10	1
*Hordeum*	14, 28, 42	14, 21, 28, 42, 63, 70	2	5.50–14.93	3.43–16.4	0
*Hygrophila*	32	(12), (24), 32, 34, 44	2			
*Hymenachne*	20	20, 40	2			
*Hyparrhenia*	20, 40	20, 30, 40, 45	2			
*Hypericum*	16, 32, 40, 48	(8), 14, 16, 18, (20), 24, 28, (30), 32, 36, 38, 40, c. 54	1	0.78	0.34–0.51	1
*Hypochaeris*	8	6, 8, 10, 12, 14, 16, 20	2	1.34	0.84–4.05	1
*Chamaecytisus*	52, (46–50)	48, 96	2	1.25	2.05–3.19	1
*Chenopodium*	18, 27, 36, 45, 54, 90	18, 36, 54	2	0.77–2.33	0.37–2.20	1
*Chloris*	20, (36)	20, 30, 40, 60	3			
*Chromolaena*	60	20, 40, 50	5			
*Ilex*	40	34, 36, 38, 40, 120	3	1.15	1.13–2.13	0
*Impatiens*	18	6, 8, 10, 12, 14, 16, 18, 20, 26, 28, 30, 32, 34, 36, 40, 50, c. 60	4	1.15	1.33–3.25	1
*Imperata*	20, 40, 60	20	1	5.43	0.73	2
*Ipomoea*	30, 60	28, 30, 32, (38), 60, 90	2	0.95	0.63–2.25	1
*Iris*	(16), (24), 34	14, 16, 18, 20, 22, 24, 26, 27, 28, 32, 34, 36, 40, 42, 44, 48, 54, 84, 108	4	5.67	4.00–28.20	1
*Jacaranda*	36	36	0	1.18	1.3	0
*Jasminum*	26, 39	24, 26, 39, 52, 65, 78	2			
*Juncus*	40, 48, 80	8, 20, 26, (28), 30, 34, 38, 40, 42, 44, (46), 48, 60, (68), 70, 80, 84, 90, 100, 106, 108, 120, 126, 132	4	0.30–1.83	0.35–1.30	0
*Lagarosiphon*				3.25	1.8	0
*Lantana*	22, 33, 44, 55, 66	22, 44, 66	1			
*Lavandula*	30	22, 30, 36, 42, 44, 48, 50, 54, 72	3			
*Leersia*	(28), 48	24, 48, 72	3			
*Lepidium*	24	(4), 16, 24, 28, 32, 48, 64	3	1.04	0.33–0.58	1
*Leucaena*	(56), 104	36, 52, 54, 56, 104, 106, 108, 110, 112	3	1.28	0.31–1.65	1
*Ligustrum*	46	46	0			
*Litsea*	24	48	5			
*Lolium*	14, (56)	14, 28	2	2.76	2.13–3.18	0
*Lonicera*	18, (36)	18, 36, 45, 54	3			
*Lotus*	12, 24, (36)	10, 12, 14, 24, 28	2	0.48–1.05	0.45–1.40	0
*Lupinus*	48, (96)	32, (34), 36, 38, 40, 42, 48, 50, 52, 54, 56, 100	3	0.85–0.90	0.49–1.34	0
*Lygodium*	58, 60	58, 60, 116	2			
*Lythrum*	30, 60	10, 20, 30	4			
*Macfadyena*	40, 80	40	1			
*Mahonia*	28, 56	28, 56	0			
*Marrubium*	34	20, 32, 34, (36)	2			
*Melaleuca*				0.97	1.13	0
*Melia*				0.43	0.48	0
*Melilotus*	16, (24)	16	1	1.23	1.13–1.33	0
*Melinis*	36	36	0			
*Mentha*	20, 30, 36, 40	18, 24, 36, 42, 48, 50, 54, 60, 66, 72, 74, 84, 90, 96, 108, 120	3			
*Miconia*	32	32, 34, 48, 50, 62, c. 134	3			
*Microstegium*	40	20, 40, 60, 70, 80	4			
*Mikania*	72	34, 36, 38, 42, 68, 108	4			
*Mimosa*	26	26, 52, 104	3			
*Montanoa*	38	38	0			
*Morus*	28, (30), 42	28, 30, 42, c. 304	2			
*Myosotis*	18, 20	18, 22, 24, 28, 32, 36, 40, 44, 48, 52, 64, 66, 72, 88	4			
*Myriophyllum*	28, (36), 42	14, 21, 28, 42	2	0.25	0.48–0.50	1
*Nephrolepis*	82	82, (164)	1			
*Neyraudia*	40	40	0			
*Nicotiana*	24, 48	18, 20, 24, 32, 34, 36, 38, 40, 42, 44, 46, 48, 60, c. 96	3	5.33	1.43–6.38	1
*Olea*				1.95	1.50–2.99	0
*Opuntia*	(12), 22, (36), 44, 55, 66	22, 33, 44, 55, 66, 77, 88	1	2.28	2.04–3.80	0
*Ornithogalum*	18, 27, 36, 44, 45, 46, 47, 51, 52, 53, 54, 55, 72, 90, 104	4, 6, 10, 12, 14, 16, 18, 20, 22, 24, 26, 27, 28, 30, 32, 34, 36, 40, 42, 46, 50, 51, 52, 54, 56, 60, 72, 76, 80, 108	2	24.85	4.10–33.45	1
*Oxalis*	24, 34	10, 12, 14, 16, 18, 22, 24, 28, 32, 35, 36, 42, 44, 48, 54, 64, 72	4	0.69–0.83	0.37–16.50	1
*Paederia*	44, 55, 66	22, 44, 66, 88	3			
*Panicum*	18, 32, 36, 40, 48	18, 20, 30, 32, 36, 40, 54, 72	2			
*Parapholis*	24, 28, 30, 36, 38	12, 14	2			
*Paspalum*	20, 30, 40, 50, 60	12, 20, 24, 30, 32, 40, 50, 60, 80	2	1.15–1.79	0.62–1.61	0
*Passiflora*	18	12, (14), 18, 20, 24, 36, 72	3	1.58	0.92–2.68	0
*Pastinaca*	22	22, (44)	1			
*Pennisetum*	18, 21, 27, 28, 36, 45, 54, 72	10, 14, 15, 16, 18, 20, 22, 28, 30, 32, 35, 36, 45, 48, 54, 56, 63, c. 70, 72, 90	2	1.15–2.90	0.85–4.80	0
*Pereskia*	22, 28	22	1			
*Petrorhagia*	30	26, 30, 60	2			
*Phalaris*	14, 28, 42	12, 14, 28, 56	2	4.13–6.45	1.38–5.70	1
*Phleum*	14, 28, 42	(10), 14, 28, 42	0	4.15	1.20–3.23	1
*Phragmites*	(28), (36), (40), 48, 54, 72, 96, 120	48, 96	1			
*Physalis*	48	24, 48, (72)	2			
*Pinus*	24	24	0	21.92–28.90	17.25–36.00	0
*Pilosella*	(18), 36, 45, 54, 63, (81), (90)	18, 27, 36, 45, 54, 63, 72	1	3.45–6.06	1.08–8.34	1
*Plantago*	10, 12, 20, 24	8, 10, 12, 20, 24, 30, 36, 48, 72, 96	3	0.86–1.20	0.50–2.78	1
*Poa*	14, 21, 28, 35, 42, 44, 48, 49, 50, 56, 58, 63, 64, 66, 70, 72, 84	14, 21, 24, 28, 33, 34, 35, 36, 39, 42, 46, 48, 49, 50, 56, 58, 63, 70, 72, 76, 80, 84, 112	1	4.24–5.38	1.18–16.28	2
*Polygonum*	20, 30, 40, 50, 60	18, 20, 22, 24, 26, 28, 30, 32, 34, 36, 40, 42, 44, 46, 48, 50, 52, 54, 58, 60, 88, 100	2	0.85	0.43–0.70	1
*Polypogon*	28	14, 28, 42, 56	4			
*Populus*	38	38, 40, 44, 45, 46, 48, 57	2	0.52	0.45–0.54	0
*Prosopis*	28, 56, 112	28, 56, 112	0	0.43–1.70	0.40–1.28	0
*Prunus*	16, 24	16, 24, 32, 48, 64	2	0.50–3.65	0.28–1.18	1
*Psidium*	22, 44	22, 44, 77	2	0.28–0.53	0.58	1
*Ranunculus*	(24), (28), 32	14, 16, 24, 28, 32, 40, 42, 48, 56, 64, 72, 80, 96, 128	4	11.2	1.90–25.10	2
*Rhamnus*	24	20, 24	1	0.31–1.33	0.25–0.56	1
*Robinia*	22	30	1			
*Romulea*	18	18, 20, 22, 24, 26, 28, 30, 32, 44, 48, 50, 56	4			
*Rorippa*	32	16, 28, 32, 40, 44, 48	3	0.54	0.19–0.73	1
*Rosa*	14, 21, 28, 35, 42	14, 21, 28, 35, 42, 49, 56	1	0.50–1.43	0.28–1.45	0
*Rubus*	14, 21, 28	14, 21, 28, 35, 42, 56, c. 70, 84, c. 98	2	0.29–0.35	0.24–1.23	1
*Rumex*	14, 20, 21, 28, 35, 40, 42, 60, c. 90	14, 15, 16, 18, 20, 22, 24, 30, 36, 40, 42, 50, 60, 80, 100, 120, 130, 140, 160, 200	2	1.68–4.40	0.48–6.24	1
*Salix*	38, 76	38, 76, 114, 152, 182, 186, c. 214	3	0.77–0.86	0.35–0.86	1
*Salsola*	36	18, 36, 54, 72	4	0.61	1.31	1
*Salvia*	(14), (16), 42, 48, 54, 56, 60, 62, 64	12, 14, 16, 8, 20, 22, 24, 26, 28, 30, 32, 40, 42, 44, 48, 52, 66, 88	2			
*Salvinia*	45	18, 36, 45, 54, 63	3			
*Sambucus*	36	(18), 36, 38, c. 72	2	15.25	10.55–10.84	1
*Securigera*	(16), 24	12, 20	3			
*Sedum*	40, 56, 60, 80, 100	10, 12, 14, 16, 18, 20, 22, 24, 26, 28, 30, 32, 34, 36, 40, 42, 44, 48, 52, 56, 58, 60, 64, 68, 70, 72, 74, 82, 84, 796, 98, 102, 104, 112, 124, 136, 140, 148, 160, 168, 185	3	1.25	0.15–9.10	2
*Senecio*	20	10, 18, 20, 24, 36, 38, 40, 44, 46, 48, 60, 70, 72, 76, 80, 90, 92, 96, 98, 100, 104, 110, 114, 120, c. 130, c. 160, 180	4			
*Senna*	26, 28	22, 24, 26, 28	1			
*Sesbania*	12	12, 14, 24	2			
*Schismus*	12	12	0			
*Schkuhria*	20, 40	20, 40	0			
*Solanum*	24, 36, 48, 60, 72	12, 16, 22, 24, 36, 48, 72, 96	3	1.33–3.10	0.63–3.65	1
*Solidago*	18, 36, 54	18, 36, 54, 90, 108	2	1.58–1.82	1.13	1
*Sonchus*	32, (64)	14, 18, 32, 36, 54	3	1.6	1.31–1.85	0
*Spartina*	60, 52, 120, 122, 124	40, 60, 62	2			
*Sporobolus*	36	18, 20, 24, 30, 36, 40, 50, 54, 60, 72	4			
*Stenotaphrum*	18, 32	18	2			
*Syzygium*	44, 66	22	5			
*Tagetes*	48	24, 48	2			
*Tamarix*	24	22, 24	1			
*Tecoma*	36	36	0			
*Tectaria*	160	80, 120, 160	2			
*Thespesia*	26	26	0	4.1	1.6	1
*Thunbergia*	56	16, 18, 28, 56	3			
*Toona*	56	52, 56	1			
*Tradescantia*	30, 40, 50, 60, 70, 108, 132, 140, 144	12, 14, 16, 18, 22, 24, 26, 28, 30, 36, 60, 72, 74, 76, 92, 114	3	4.05–12.93	4.25–43.35	1
*Trifolium*	14, 16, 28, 32	10, 12, 14, 16, 24, 32, 48, 56, 60, 64, 80, 84, 96, 126	4	0.39–1.12	0.34–5.79	1
*Typha*	30	30, 60	2			
*Ulex*	32, 64, 96	32, 64, 96	0	3.85	2.9	0
*Ulmus*	28	28, 56	2			
*Urochloa*	36	28, 30, 36, 48	3			
*Verbascum*	36, c. 64	26, (28), 30, 32, 36, 44, 46, 56	2			
*Verbena*	28	10, 14, 20, 28, 42	3			
*Vinca*	90, 92	16, 46, (92)	4	2.1	0.74–0.76	2
*Vulpia*	14, 28, 42	14, 28, 35, 42	1	2.93–6.89	2.30–4.41	0
*Watsonia*	18, 27	18, (20), 27	1			
*Xanthium*	36	36	0	3.15	2.59	0
*Zantedeschia*	32	32	0	2.3	2.3	0
*Zizania*	20, 34	30	2			
*Ziziphus*	48	22, 24, 36, 48	3			

Invasive species are classified as those listed in Weber (2003), and non-invasive as those not included in that list (each chromosome number datum refers to a separate species). If related congeners are morphologically similar but differ in invasiveness that is related to ploidy levels, then misidentification based on morphology would result in swapping their karyological data, and lead to spurious results in ecological studies. Ploidy variation data are based on chromosome numbers (Goldblatt and Johnson 1979 onwards), and genome size data represent holoploid genome size values (taken from Bennett and Leitch 2012). Values presented in parentheses are either dubious or not found *in situ*. The risk categories are defined as follows. For chromosome numbers: 0—karyologically homogeneous genus, 1—largely corresponding chromosome numbers between invasive and non-invasive species, 2—slightly different chromosome numbers between invasive and non-invasive species (inter-group difference between extreme chromosome numbers less than 2-fold), 3—different chromosome numbers between invasive and non-invasive species (chromosomal variation of one group usually constitutes a fraction of the variation observed in the other group, inter-group difference between extreme chromosome numbers at least 2-fold at one end), 4—considerably different chromosome numbers between invasive and non-invasive species (chromosomal variation of one group usually constitutes only a small fraction of the variation observed in the other group, inter-group difference between extreme chromosome numbers at least 2-fold at both ends), 5—mutually exclusive chromosome numbers between invasive and non-invasive species. For genome size: 0—low difference in genome size between invasive and non-invasive species (inter-group difference between extreme C-values less than 2-fold at any end), 2—certain differences in genome size between invasive and non-invasive species (inter-group difference between extreme C-values at least 2-fold at one end), 3—high differences in genome size between invasive and non-invasive species (inter-group difference between extreme C-values at least 2-fold at both ends or mutually exclusive C-values).

## References

[PLT042C1] Abbott RJ (1992). Plant invasions, interspecific hybridization and the evolution of new plant taxa. Trends in Ecology & Evolution.

[PLT042C2] Abbott RJ, James JK, Milne RI, Gillies ACM (2003). Plant introductions, hybridization and gene flow. Philosophical Transactions of the Royal Society B.

[PLT042C3] Agnarsson I, Kuntner M (2007). Taxonomy in a changing world: seeking solutions for a science in crisis. Systematic Biology.

[PLT042C4] Aikio S, Duncan RP, Hulme PE (2010). Lag-phases in alien plant invasions: separating the facts from the artefacts. Oikos.

[PLT042C5] Aikio S, Duncan RP, Hulme PE (2012). The vulnerability of habitats to plant invasion: disentangling the roles of propagule pressure, time and sampling effort. Global Ecology & Biogeography.

[PLT042C6] Ainouche ML, Fortune M, Salmon A, Parisod C, Grandbastien MA, Fukunaga K, Ricou M, Missel MT (2009). Hybridization, polyploidy and invasion: lessons from *Spartina* (Poaceae). Biological Invasions.

[PLT042C7] Amsellem L, Noyer JL, Le Bourgeois T, Hossaert-McKey M (2000). Comparison of genetic diversity of the invasive weed *Rubus alceifolius* Poir. (Rosaceae) in its native range and in areas of introduction, using amplified fragment length polymorphism (AFLP) markers. Molecular Ecology.

[PLT042C8] Angiosperm Phylogeny Group (2009). An update of the Angiosperm Phylogeny Group classification for the orders and families of flowering plants: APG III. Botanical Journal of the Linnean Society.

[PLT042C9] Anttila CK, Daehler CC, Rank NE, Strong DR (1998). Greater male fitness of a rare invader (*Spartina alterniflora*, Poaceae) threatens a common native (*Spartina foliosa*) with hybridization. American Journal of Botany.

[PLT042C10] Ayres DR, Grotkopp EK, Zaremba K, Sloop CM, Blum MJ, Bailey JP, Anttila CK, Strong DM (2008). Hybridization between invasive *Spartina densiflora* (Poaceae) and native *S. foliosa* in San Francisco Bay, California, USA. American Journal of Botany.

[PLT042C11] Baamonde S, Baspino I, Barreiro R, Cremades J (2007). Is the cryptic alien seaweed *Ulva pertusa* (Ulvales, Chlorophyta) widely distributed along European Atlantic coasts?. Botanica Marina.

[PLT042C12] Bean AR (2007). A new system for determining which plant species are indigenous in Australia. Australian Systematic Botany.

[PLT042C13] Bennett MD, Leitch IJ (2012). http://data.kew.org/cvalues/.

[PLT042C14] Blackburn TM, Pyšek P, Bacher S, Carlton JT, Duncan RP, Jarošík V, Wilson JRU, Richardson DM (2011). A proposed unified framework for biological invasions. Trends in Ecology and Evolution.

[PLT042C15] Briese DT, Walker A (2008). Choosing the right plants to test: the host-specificity of *Longitarsus* sp. (Coleoptera: Chrysomelidae) a potential biological control agent of *Heliotropium amplexicaule*. Biological Control.

[PLT042C16] Browning J, Gordon-Gray KD (2000). Patterns of fruit morphology in *Bolboschoenus* (Cyperaceae) and their global distribution. South African Journal of Botany.

[PLT042C17] Browning J, Gordon-Gray KD, Smith GS (1995). Achene structure and taxonomy of North American *Bolboschoenus* (Cyperaceae). Brittonia.

[PLT042C18] Cadotte MW, Murray BR, Lovett-Doust J (2006). Ecological patterns and biological invasions: using regional species inventories in macroecology. Biological Invasions.

[PLT042C19] CBOL Plant Working Group (2009). A DNA barcode for land plants. Proceedings of the National Academy of Sciences of the USA.

[PLT042C20] Chapman H, Robson B, Pearson ML (2004). Population genetic structure of a colonising, triploid weed, *Hieracium lepidulum*. Heredity.

[PLT042C21] Chase MW, Fay MF (2009). Barcoding of plants and fungi. Science.

[PLT042C22] Chu D, Guo D, Pan H, Zhang Y, Wan F (2012). Cryptic invasion of alien species: types and effects. Acta Entomologica Sinica.

[PLT042C23] Clark BR, Godfray HCJ, Kitching IJ, Mayo SJ, Scoble MJ (2009). Taxonomy as eScience. Transactions of the Royal Society of London A.

[PLT042C24] Cleland RE (1972). Oenothera cytogenetics and evolution.

[PLT042C25] Costello MJ, May RM, Stork NE (2013). Can we name Earth's species before they go extinct?. Science.

[PLT042C26] Cross HB, Lowe AJ, Gurgel CF, Richardson DM (2011). DAN barcoding of invasive species. Fifty years of invasion ecology. The legacy of Charles Elton.

[PLT042C27] Daehler CC, Strong DR (1994). Variable reproductive output among clones of *Spartina alterniflora* (Poaceae) including San Francisco Bay, California: the influence of herbivory, pollination and establishment rate. American Journal of Botany.

[PLT042C28] DAISIE (2009). Handbook of alien species in Europe.

[PLT042C29] Danihelka J, Chrtek J, Kaplan Z (2012). Checklist of vascular plants of the Czech Republic. Preslia.

[PLT042C30] De Filipps RA, Tutin TG, Heywood N, Burges A, Moore DM, Valentine DH, Walter SM, Webb DA (1980). *Scirpus* L. Flora Europaea.

[PLT042C31] de Queiroz K (2005). Ernst Mayr and the modern concept of species. Proceedings of the National Academy of Sciences of the USA.

[PLT042C32] de Queiroz K (2007). Species concepts and species delimitation. Systematic Biology.

[PLT042C33] Dietrich W, Wagner WL, Raven PH (1997). Systematics of *Oenothera* section *Oenothera* subsection *Oenothera* (Onagraceae). Systematic Botany Monographs.

[PLT042C34] Ellstrand NC, Schierenbeck KA (2000). Hybridization as a stimulus for the evolution of invasiveness in plants?. Proceedings of the National Academy of Sciences of the USA.

[PLT042C35] Ensing DJ, Moffat CE, Pither J (2013). Taxonomic identification errors generate misleading ecological niche model predictions of an invasive hawkweed. Botany.

[PLT042C36] Essl F, Dullinger S, Rabitsch W, Hulme PE, Hülber K, Jarošík V, Kleinbauer I, Krausmann F, Kühn I, Nentwig W, Vilà M, Genovesi P, Gherardi F, Desprez-Lousteau M-L, Roques A, Pyšek P (2011). Socioeconomic legacy yields an invasion debt. Proceedings of the National Academy of Sciences of the USA.

[PLT042C37] Fuentes N, Kühn I, Ugarte E, Klotz S (2008). Alien plants in Chile. Inferring invasion periods from herbarium records. Biological Invasions.

[PLT042C38] Gaskin JF, Schaal BA (2002). Hybrid *Tamarix* widespread in US invasion and undetected in native Asian range. Proceedings of the National Academy of Sciences of the USA.

[PLT042C39] Ghahramanzadeh R, Esselink G, Kodde LP, Duistermaat H, van Valkenburg JLCH, Marashi SH, Smulders MJM, van de Wiel CCM (2013). Efficient distinction of invasive aquatic plant species from non-invasive related species using DNA barcoding. Molecular Ecology Resources.

[PLT042C40] Godfray HJC (2002). Challenges for taxonomy: the discipline will have to reinvent itself if it is to survive and flourish. Nature.

[PLT042C41] Godfray HCJ (2005). Taxonomy as information science. Proceedings of the Californian Academy of Science.

[PLT042C42] Godfray HCJ (2007). Linnaeus in the information age. Nature.

[PLT042C43] Godfray HCJ, Knapp S (2004). Introduction to theme issue, ‘Taxonomy for the 21st Century. Philosophical Transactions of the Royal Society, London B.

[PLT042C44] Godfray HCJ, Clark BR, Kitching IJ, Mayo SJ, Scoble MJ (2008a). The web and the structure of taxonomy. Systematic Biology.

[PLT042C45] Godfray HCJ, Mayo SJ, Scoble MJ (2008b). Pragmatism and rigour can coexist in taxonomy. Evolutionary Biology.

[PLT042C46] Goldblatt P, Johnson DE (1979). Index to plant chromosome numbers.

[PLT042C47] Goolsby JA, De Barro PJ, Makinson JR, Pemberton RW, Hartley DM, Frohlich DR (2006). Matching the origin of an invasive weed for selection of a herbivore haplotype for a biological control programme. Molecular Ecology.

[PLT042C48] Gotelli NJ (2004). A taxonomic wish-list for community ecology. Philosophical Transactions of the Royal Society of London B.

[PLT042C49] Graham CH, Ferrier S, Huettman F, Moritz C, Peterson AT (2004). New developments in museum-based informatics and applications in biodiversity analysis. Trends in Ecology and Evolution.

[PLT042C50] Grotkopp E, Rejmánek M, Sanderson MJ, Rost TL (2004). Evolution of genome size in pines (*Pinus*) and its life-history correlates: supertree analyses. Evolution.

[PLT042C51] Guo W-Y, Lambertini C, Li X-Z, Meyerson LA, Brix H (2013). Invasion of Old World *Phragmites australis* in the New World: precipitation and human influences redesign the invasive niche. Global Change Biology.

[PLT042C52] Hey J (2006). On the failure of modern species concepts. Trends in Ecology and Evolution.

[PLT042C53] Hollingsworth PM (2011). Refining the DNA barcode for land plants. Proceedings of the National Academy of Sciences of the USA.

[PLT042C54] Hovick SM, Campbell LG, Snow AA, Whitney KD (2012). Hybridization alters early life-history traits and increases plant colonization success in a novel region. American Naturalist.

[PLT042C55] Hroudová Z, Zákravský P, Ducháček M, Marhold K (2007). Taxonomy, distribution and ecology of *Bolboschoenus* in Europe. Annales Botanici Fennici.

[PLT042C56] Hughes KA, Convey P (2012). Determining the native/non-native status of newly discovered terrestrial and freshwater species in Antarctica—current knowledge, methodology and management action. Journal of Environmental Management.

[PLT042C57] Hulme PE, Hester R, Harrison RM (2007). Biological invasions in Europe: drivers, pressures, states, impacts and responses biodiversity under threat. Issues in environmental science and technology.

[PLT042C58] Hulme PE (2011). Addressing the threat to biodiversity from botanic gardens. Trends in Ecology and Evolution.

[PLT042C60] Hulme PE, Weser C (2011). Mixed messages from multiple information sources on invasive species: a case of too much of a good thing?. Diversity and Distributions.

[PLT042C59] Hulme PE, Barrett SCH (2013). Integrating trait- and niche-based approaches to assess contemporary evolution in alien plant species. Journal of Ecology.

[PLT042C61] Hulme PE, Nentwig W, Pyšek P, Vilà M (2009). Common market, shared problems: time for a coordinated response to biological invasions in Europe?. Neobiota.

[PLT042C62] Jahodová Š, Trybush S, Pyšek P, Wade M, Karp A (2007). Invasive species of *Heracleum* in Europe: an insight into genetic relationships and invasion history. Diversity and Distributions.

[PLT042C63] Jimenez-Valverde A, Lira-Noriega A, Peterson AT, Soberon J (2010). Marshalling existing biodiversity data to evaluate biodiversity status and trends in planning exercises. Ecological Research.

[PLT042C64] Joppa LN, Roberts DL, Pimm SL (2011). The population ecology and social behaviour of taxonomists. Trends in Ecology and Evolution.

[PLT042C65] Kettenring KM, Mock KE (2012). Genetic diversity, reproductive mode, and dispersal differ between the cryptic invader, *Phragmites australis*, and its native conspecific. Biological Invasions.

[PLT042C66] Kirschner J, Kaplan Z (2002). Taxonomic monographs in relation to global Red Lists. Taxon.

[PLT042C67] Kramer AT, Zorn-Arnold B, Havens K (2010). Assessing botanical capacity to address grand challenges in the United States.

[PLT042C68] Krell F-T (2000). Impact factors aren't relevant to taxonomy. Nature.

[PLT042C69] Krell F-T (2002). Why impact factors don't work for taxonomy. Nature.

[PLT042C70] Kubešová M, Moravcová L, Suda J, Jarošík V, Pyšek P (2010). Naturalized plants have smaller genomes than their non-invading relatives: a flow cytometric analysis of the Czech alien flora. Preslia.

[PLT042C71] Lambdon PW, Pyšek P, Basnou C, Hejda M, Arianoutsou M, Essl F, Jarošík V, Pergl J, Winter M, Anastasiu P, Andriopoulos P, Bazos I, Brundu G, Celesti-Grapow L, Chassot P, Delipetrou P, Josefsson M, Kark S, Klotz S, Kokkoris Y, Kühn I, Marchante H, Perglová I, Pino J, Vilà M, Zikos A, Roy D, Hulme PE (2008). Alien flora of Europe: species diversity, temporal trends, geographical patterns and research needs. Preslia.

[PLT042C72] Lambertini C, Sorrell BK, Riis T, Olesen B, Brix H (2012). Exploring the borders of European *Phragmites* within a cosmopolitan genus. AoB PLANTS.

[PLT042C73] Lavergne S, Muenke NJ, Molofsky J (2010). Genome size reduction can trigger rapid phenotypic evolution in invasive plants. Annals of Botany.

[PLT042C74] Leimu R, Koricheva J (2005). What determines the citation frequency of ecological papers?. Trends in Ecology and Evolution.

[PLT042C75] Leitch IJ, Bennett MD, Doležel J, Greilhuber J, Suda J (2007). Genome size and its uses: the impact of flow cytometry. Flow cytometry with plant cells. Analysis of genes, chromosomes and genomes.

[PLT042C76] Levin DA (2002). The role of chromosomal change in plant evolution.

[PLT042C77] Mandák B, Pyšek P, Lysák M, Suda J, Krahulcová A, Bímová K (2003). Variation in DNA-ploidy levels of *Reynoutria* taxa in the Czech Republic. Annals of Botany.

[PLT042C78] McGeoch MA, Spear D, Kleynhans EJ, Marais E (2012). Uncertainty in invasive alien species listing. Ecological Applications.

[PLT042C79] Meyer CP, Paulay G (2005). DNA barcoding: error rates based on comprehensive sampling. PLoS Biology.

[PLT042C80] Meyerson LA, Cronin JT (2013). Evidence for multiple introductions of *Phragmites australis* to North America: detection of a new non-native haplotype. Biological Invasions.

[PLT042C81] Meyerson LA, Saltonstall K, Chambers RM, Silliman BR, Grosholz E, Bertness MD (2009). *Phragmites australis* in eastern North America: a historical and ecological perspective. Salt marshes under global Siege.

[PLT042C82] Meyerson LA, Lambert AM, Saltonstall K (2010a). Three invasion fronts of *Phragmites australis* in North America: research and management needs in the face of common reed expansion in the west and Gulf Regions. Invasive Plant Science and Management.

[PLT042C83] Meyerson LA, Viola D, Brown RN (2010b). Hybridization of invasive *Phragmites australis* with a native subspecies in North America. Biological Invasions.

[PLT042C84] Meyerson LA, Lambertini C, McCormick MK, Whigham DF (2012). Hybridization of common reed in North America? The answer is blowing in the wind. AoB PLANTS.

[PLT042C85] Mihulka S, Pyšek P (2001). Invasion history of *Oenothera* congeners in Europe: a comparative study of spreading rates in the last 200 years. Journal of Biogeography.

[PLT042C86] Milne RI, Abbott RJ (2000). Origin and evolution of invasive naturalised material of *Rhododendron ponticum* L. in the British Isles. Molecular Ecology.

[PLT042C87] Moody ML, Les DH (2002). Evidence of hybridity in invasive watermilfoil (*Myriophyllum*) populations. Proceedings of the National Academy of Sciences of the USA.

[PLT042C88] Novak SJ, Mack RN (2000). Clonal diversity within and among introduced populations of the apomictic vine *Bryonia alba* (Cucurbitaceae). Canadian Journal of Botany.

[PLT042C89] Pandit MK, Pocock MJO, Kunin WE (2011). Ploidy influences rarity and invasiveness in plants. Journal of Ecology.

[PLT042C90] Paterson ID, Hoffmann JH, Klein H, Mathenge CW, Neser S, Zimmermann HG (2011). Biological control of Cactaceae in South Africa. African Entomology.

[PLT042C91] Patterson DJ, Cooper J, Kirk PM, Pyle RL, Remsen DP (2010). Names are key to the big new biology. Trends in Ecology and Evolution.

[PLT042C92] Paul J, Vachon N, Garroway CJ, Freeland JR (2010). Molecular data provide strong evidence of natural hybridization between native and introduced lineages of *Phragmites australis* in North America. Biological Invasions.

[PLT042C93] Petit RJ (2004). Biological invasions at the gene level. Diversity and Distributions.

[PLT042C94] Piredda R, Simeone MC, Attimonelli M, Bellarosa R, Schirone B (2011). Prospects of barcoding the Italian wild dendroflora: oaks reveal severe limits to track species identity. Molecular Ecology Resources.

[PLT042C95] Pires AC, Marinoni L (2010). DNA barcoding and traditional taxonomy unified through integrative taxonomy: a view that challenges the debate questioning both methodologies. Biota Neotropica.

[PLT042C96] Pyšek P (2003). How reliable are data on alien species in Flora Europaea?. Flora.

[PLT042C97] Pyšek P, Hulme PE (2005). Spatio-temporal dynamics of plant invasions: Linking pattern to process. Ecoscience.

[PLT042C98] Pyšek P, Richardson DM (2010). Invasive species, environmental change and management, and health. Annual Review of Environment and Resources.

[PLT042C99] Pyšek P, Mandák B, Francírková T, Prach K, Brundu G, Brock J, Camarda I, Child L, Wade M (2001). Persistence of stout clonal herbs as invaders in the landscape: a field test of historical records. Plant invasions: species ecology and ecosystem management.

[PLT042C100] Pyšek P, Sádlo J, Mandák B (2002). Catalogue of alien plants of the Czech Republic. Preslia.

[PLT042C101] Pyšek P, Brock JH, Bímová K, Mandák B, Jarošík V, Koukolíková I, Pergl J, Štěpánek J (2003). Vegetative regeneration in invasive *Reynoutria* (Polygonaceae) taxa: the determinant of invasibility at the genotype level. American Journal of Botany.

[PLT042C102] Pyšek P, Richardson DM, Rejmánek M, Webster G, Williamson M, Kirschner J (2004). Alien plants in checklists and floras: towards better communication between taxonomists and ecologists. Taxon.

[PLT042C103] Pyšek P, Richardson DM, Pergl J, Jarošík V, Sixtová Z, Weber E (2008). Geographical and taxonomic biases in invasion ecology. Trends in Ecology and Evolution.

[PLT042C104] Pyšek P, Jarošík V, Pergl J, Randall R, Chytrý M, Kühn I, Tichý L, Danihelka J, Chrtek J, Sádlo J (2009). The global invasion success of Central European plants is related to distribution characteristics in their native range and species traits. Diversity and Distribution.

[PLT042C105] Pyšek P, Jarošík V, Hulme PE, Kühn I, Wild J, Arianoutsou M, Bacher S, Chiron F, Didžiulis V, Essl F, Genovesi P, Gherardi F, Hejda M, Kark S, Lambdon PW, Desprez-Loustau A-M, Nentwig W, Pergl J, Poboljšaj K, Rabitsch W, Roques A, Roy DB, Shirley S, Solarz W, Vilà M, Winter M (2010). Disentangling the role of environmental and human pressures on biological invasions across Europe. Proceedings of the National Academy of Sciences of the USA.

[PLT042C106] Pyšek P, Chytrý M, Pergl J, Sádlo J, Wild J (2012a). Plant invasions in the Czech Republic: current state, introduction dynamics, invasive species and invaded habitats. Preslia.

[PLT042C107] Pyšek P, Danihelka J, Sádlo J, Chrtek J, Chytrý M, Jarošík V, Kaplan Z, Krahulec F, Moravcová L, Pergl J, Štajerová K, Tichý L (2012b). Catalogue of alien plants of the Czech Republic (2nd edition): checklist update, taxonomic diversity and invasion patterns. Preslia.

[PLT042C108] Richardson DM, Pyšek P, Rejmánek M, Barbour MG, Panetta FD, West CJ (2000). Naturalization and invasion of alien plants: concepts and definitions. Diversity and Distributions.

[PLT042C109] Richardson DM, Carruthers J, Hui C, Impson FAC, Robertson MP, Rouget M, Le Roux JJ, Wilson JRU (2011). Human-mediated introductions of Australian acacias: a global experiment in biogeography. Diversity and Distributions.

[PLT042C110] Robertson MP, Cumming GS, Erasmus BFN (2010). Getting the most out of atlas data. Diversity and Distribution.

[PLT042C111] Rocchini D, Hortal J, Lengyel S, Lobo JM, Jimenez-Valverde A, Ricotta C, Bacaro G, Chiarucci A (2011). Accounting for uncertainty when mapping species distributions: the need for maps of ignorance. Progress in Physical Geography.

[PLT042C112] Saltonstall K (2002). Cryptic invasion by a non-native genotype of the common reed, *Phragmites australis*, into North America. Proceedings of the National Academy of Sciences of the USA.

[PLT042C149] Saltonstall K, Peterson PM, Soreng RJ (2004). Recognition of *Phragmites australis* subsp. *americanus* (Poaceae: Arundinoideae) in North America: evidence from morphological and genetic analyses. Sida.

[PLT042C113] Santos AM, Branco M (2012). The quality of name-based species records in databases. Trends in Ecology and Evolution.

[PLT042C114] Sax DF (2001). Latitudinal gradients and geographic ranges of exotic species: implications for biogeography. Journal of Biogeography.

[PLT042C115] Schierenbeck KA, Ellstrand NC (2009). Hybridization and the evolution of invasiveness in plants and other organisms. Biological Invasions.

[PLT042C116] Scott WA, Hallam CJ (2002). Assessing species misidentification rates through quality assurance of vegetation monitoring. Plant Ecology.

[PLT042C117] Seifert K, Crous P, Frisvad J (2008). Correcting the impact factors of taxonomic journals by ACT. Inoculum.

[PLT042C118] Shen Y-Y, Chen X, Murphy RW (2013). Assessing DNA barcoding as a tool for species identification and data quality control. PLoS One.

[PLT042C119] Shine C, Williams N, Gundling L (2000). A guide to designing legal and institutional frameworks on alien invasive species.

[PLT042C120] Smith GF, Figueiredo E (2007). Naturalized species of *Agave* L. (Agavaceae) on the southeastern coast of Portugal. Haseltonia.

[PLT042C121] Smith GF, Figueiredo E (2009). Capacity building in taxonomy and systematics. Taxon.

[PLT042C122] Smith RD, Aradottir GI, Taylor A, Lyal C (2008a). Invasive species management: what taxonomic support is needed?.

[PLT042C123] Smith GF, Buys M, Walters M, Herbert D, Hamer M (2008b). Taxonomic research in South Africa: the state of the discipline. South African Journal of Science.

[PLT042C124] Soltis DE, Soltis PS (1999). Polyploidy: recurrent formation and genome evolution. Trends in Ecology and Evolution.

[PLT042C125] Stuessy TF, Lack HW (2011). Monographic plant systematics: fundamental assessment of plant biodiversity.

[PLT042C126] Suda J, Trávníček P, Mandák B, Berchová-Bímová K (2010). Genome size as a marker for identifying the invasive alien taxa in *Fallopia* section *Reynoutria*. Preslia.

[PLT042C127] Tavalire HF, Bugbee GE, LaRue EA, Thum RA (2012). Hybridization, cryptic diversity, and invasiveness in introduced variable-leaf watermilfoil. Evolutionary Applications.

[PLT042C128] te Beest M, Le Roux JJ, Richardson DM, Brysting AK, Suda J, Kubešová M, Pyšek P (2012). The more the better? The role of polyploidy in facilitating plant invasions. Annals of Botany.

[PLT042C130] Thompson GD, Robertson MP, Webber BL, Richardson DM, Le Roux JJ, Wilson JRU (2011). Predicting the sub-specific identity of invasive species using distribution models: *Acacia saligna* as an example. Diversity and Distributions.

[PLT042C129] Thompson JD (1991). The biology of an invasive plant. What makes *Spartina anglica* so successful?. BioScience.

[PLT042C131] Treier UA, Broennimann O, Normand S, Guisan A, Schaffner U, Steinger T, Müller-Schärer H (2009). Shift in cytotype frequency and niche space in the invasive plant *Centaurea maculosa*. Ecology.

[PLT042C132] Tutin TG, Heywood VH, Burges NA, Moore DM, Valentine DH, Walters SM, Webb DA (1964–1980). Flora Europaea.

[PLT042C133] Valdecasas AG, Castroviejo S, Marcus LF (2000). Reliance on the citation index undermines the study of biodiversity. Nature.

[PLT042C134] Valentini A, Pompanon F, Taberlet P (2008). DNA barcoding for ecologists. Trends in Ecology and Evolution.

[PLT042C135] Venette RC, Kriticos DJ, Magarey RD, Koch FH, Baker RHA, Worner SP, Gomez Raboteaux NN, McKenney DW, Dobesberger EJ, Yemshanov D, De Barro PJ, Hutchison WD, Fowler G, Kalaris TM, Pedlar J (2010). Pest risk maps for invasive alien species: a roadmap for improvement. BioScience.

[PLT042C136] Verloove F (2010). Invaders in disguise. Conservation risks derived from misidentifications of invasive plants. Management of Biological Invasions.

[PLT042C137] Verloove F, Lambinon J (2008). Neophytes in Belgium: corrections and adjustments. Systematics and Geography of Plants.

[PLT042C138] Vilà M, D'Antonio CM (1998). Hybrid vigor for clonal growth in *Carpobrotus* (Aizoaceae) in coastal California. Ecological Applications.

[PLT042C139] Vilà M, Weber E, D'Antonio CM (2000). Conservation implications of invasion by plant hybridization. Biological Invasions.

[PLT042C140] Wapshere A (1974). A strategy for evaluating the safety of organisms for biological weed control. Annals of Applied Biology.

[PLT042C141] Weber E (2003). Invasive plant species of the world: a reference guide to environmental weeds.

[PLT042C142] Wheeler QD, Raven PH, Wilson EO (2004). Taxonomy: impediment or expedient?. Science.

[PLT042C144] Wilson JRU, Richardson DM, Rouget M, Procheş Ş, Amis MA, Henderson L, Thuiller W (2007). Residence time and potential range: crucial considerations in modelling plant invasions. Diversity and Distributions.

[PLT042C145] Wilson JRU, Ivey P, Manyama P, Nänni I (2013). A new national unit for invasive species detection, post-border risk assessment, and eradication planning. South African Journal of Science.

[PLT042C143] Wilson RL, Hoch WA (2009). Identification of sterile, noninvasive cultivars of Japanese spirea. HortScience.

[PLT042C146] Winter M, Schweiger O, Klotz S, Nentwig W, Andriopoulos P, Arianoutsou M, Basnou C, Delipetrou P, Didžiulis V, Hejda M, Hulme PE, Lambdon PW, Pergl J, Pyšek P, Roy DB, Kühn I (2009). Plant extinctions and introductions lead to phylogenetic and taxonomic homogenization of the European flora. Proceedings of the National Academy of Sciences of the USA.

[PLT042C147] Wittenberg R, Cock MJW (2001). Invasive alien species: a toolkit for best prevention and management practices.

[PLT042C148] Zhang W, Fan X, Zhu S, Zhao H, Fu L (2013). Species-specific identification from incomplete sampling: applying DNA barcodes to monitoring invasive *Solanum* plants. PLoS One.

